# Multiple bHLH Proteins form Heterodimers to Mediate CRY2-Dependent Regulation of Flowering-Time in *Arabidopsis*

**DOI:** 10.1371/journal.pgen.1003861

**Published:** 2013-10-10

**Authors:** Yawen Liu, Xu Li, Kunwu Li, Hongtao Liu, Chentao Lin

**Affiliations:** 1 National Key Laboratory of Plant Molecular Genetics, Institute of Plant Physiology and Ecology, Shanghai Institutes for Biological Sciences, Chinese Academy of Sciences, Shanghai, China; 2 Department of Molecular, Cell and Developmental Biology, University of California, Los Angeles, Los Angeles, California, United States of America; University of California Berkeley and USDA/ARS, United States of America

## Abstract

*Arabidopsis thaliana* cryptochrome 2 (CRY2) mediates light control of flowering time. CIB1 (CRY2-interacting bHLH 1) specifically interacts with CRY2 in response to blue light to activate the transcription of *FT* (*Flowering Locus T*). *In vitro*, CIB1 binds to the canonical E-box (CACGTG, also referred to as G-box) with much higher affinity than its interaction with non-canonical E-box (CANNTG) DNA sequences. However, *in vivo*, CIB1 binds to the chromatin region of the *FT* promoter, which only contains the non-canonical E-box sequences. Here, we show that CRY2 also interacts with at least CIB5, in response to blue light, but not in darkness or in response to other wavelengths of light. Our genetic analysis demonstrates that CIB1, CIB2, CIB4, and CIB5 act redundantly to activate the transcription of *FT* and that they are positive regulators of CRY2 mediated flowering. More importantly, CIB1 and other CIBs proteins form heterodimers, and some of the heterodimers have a higher binding affinity than the CIB homodimers to the non-canonical E-box in the *in vitro* DNA-binding assays. This result explains why *in vitro* CIB1 and other CIBs bind to the canonical E-box (G-box) with a higher affinity, whereas they are all associated with the non-canonical E-boxes at the *FT* promoter *in vivo*. Consistent with the hypothesis that different CIB proteins play similar roles in the CRY2-midiated blue light signaling, the expression of CIB proteins is regulated specifically by blue light. Our study demonstrates that CIBs function redundantly in regulating CRY2-dependent flowering, and that different CIBs form heterodimers to interact with the non-canonical E-box DNA *in vivo*.

## Author Summary

*Arabidopsis thaliana* blue light receptor cryptochromes (CRYs) mediate light control of flowering time by interacting with CIB1 (CRY2-interacting bHLH1) in response to blue light. However, it remains unclear how the blue light-dependent CRY2-CIB1 interaction affects the *FT* transcription. We report here that in addition to CIB1, CRY2 also interact with CIB1 related bHLH proteins, CIBs. These CIBs act redundantly with CIB1 to activate the transcription of *FT* and flowering. More importantly, CIB1 and the CIBs can form heterodimers and some of those heterodimers have a higher binding affinity to the non-canonical E-box, although their homodimers all prefer canonical E-box (G-box), so they can bind to the non-canonical E-Box sequences of the *FT* promoter. This is the first example in plants that heterodimerization of bHLH can change the DNA binding affinity or specificity. CIB proteins are involved in blue light signaling and they are specifically stabilized by blue light.

## Introduction

Cryptochromes are photolyase-like photoreceptors regulating photomorphogenesis in plants and the circadian clock in plants and animals [1]–[4]. The *Arabidopsis thaliana* genome encodes at least two cryptochromes, cryptochrome 1 (CRY1) and cryptochrome 2 (CRY2). The major function of CRY1 is to mediate blue light-dependent de-etiolation responses [5], whereas CRY2 mediates primarily photoperiodic regulation of floral initiation [6]. Cryptochromes may mediate photoperiodic control of floral initiation by at least three different mechanisms: 1. Cryptochromes mediate light suppression of the COP1-dependent degradation of *CONSTANS* (CO) [7]–[9], which is a major transcription regulator of floral initiation. CO is a critical positive regulator of flowering in long day condition, CO promotes the flowering initiation by activating transcription of the florigen gene *FT* [10], which encodes a mobile transcriptional regulator that migrates from leaves to the apical meristem to activate transcription of floral meristem identity genes [11], [12]. 2. Cryptochromes regulate the light entrainment of the circadian clock [13], and then affect the expression of *CO*. 3. Cryptochromes directly modulate the transcription of *FT* through interaction with CIB1, a basic-helix-loop-helix (bHLH) transcription factor, which was isolated in a blue light differentiated yeast-two-hybrid screen [14].

In *Arabidopsis*, at least three types of photoreceptors, crypto!chromes, the LOV-domain/F-box proteins FKF/ZTL, and phytochromes, are involved in the control of overlapping physiological functions essential to plant development, such as de-etiolation and photoperiodic flowering. Direct interaction between photoreceptors and their respective target proteins have been recognized as a fundamental mechanism underlying the signal transduction of those photoreceptors. Light-dependent protein-protein interaction has been demonstrated for phytochromes, FKF/ZTL and cryptochromes. For example, phytochromes interact with several target proteins with a wavelength preference, including a nucleoside diphosphate kinase (NDPK2), a protein phosphotase (PAPPs), a response regulator (ARR4), and several bHLH transcription factors (PIF proteins), to modulate phytochrome function and regulation [15]–[21]. The FMN-containing blue light receptors, FKF1 and ZTL, interact with a clock protein, GI, in a blue light-dependent manner to control the stability of their targets, CDF and TOC1, as well as the circadian rhythmic transcription and photoperiodic flowering [22]–[26]. Similarly, *Arabidopsis* CRY2 undergoes blue light-specific interaction with CIB1 and also SPA1 [9], [14].

*Arabidopsis* CIB1 is the first blue light-dependent CRY2-interacting protein identified in plants [14], [27], [28]. CIB1 positively regulates floral initiation in a CRY2-dependent manner. CIB1 binds to the canonical E-box (CACGTG, G-box) *in vitro* with a much higher affinity than to non-canonical E-box elements (CANNTG), but it appears to affect transcription, with similar activities, of promoters containing canonical or non-canonical E-box *in vivo*. It was shown in a transient *Arabidopsis* transcription assay that CIB1 acted as a CRY- and blue light-dependent transcription regulator, and the *in vivo* transcriptional regulation activity of CIB1 seems indiscriminatory toward canonical and non-canonical E-boxes. CIB1 stimulates *FLOWERING LOCUS T (FT)* messenger RNA expression. It interacts with the chromatin DNA of the *FT* gene that lacks a canonical E-box but contains various non-canonical E-box elements. These results suggest a significant difference between the CIB1 DNA-binding activity *in vitro* and its transcription regulatory activity *in vivo*. One possible interpretation of this dilemma would be that CIB1 heterodimerizes with other bHLH proteins to alter their preference or affinity to different DNA sequences *in vivo*.

In this study, we performed a systematic biochemical and genetic analysis to isolate additional members of the bHLH family related to CIB1, and found that at least three additional CIB1-related bHLH proteins, referred to as CIB2, CIB4, and CIB5, can interact with CRY2 and/or CIB1. CIBs function redundantly to activate the transcription of *FT* and flowering initiation. More importantly, when added individually *in vitro* they all exhibit higher binding affinity for the canonical E-box (G-box), but they undergo a switch in preference for the non-canonical E-box of the *FT* promoter when combined. This is presumably due to a switch from homodimerization to heterodimerization. These results suggest that multiple CIB proteins act redundantly in the CRY2-CIB signal transduction pathway to mediate promotion of floral initiation. Consistent with our hypothesis, CIBs are specifically involved in CRY2 signaling, the expression of CIBs proteins is regulated specifically by blue light.

## Results

### Multiple bHLH proteins demonstrate CRY2-dependent activity of promoting floral initiation

**Figure 1:**
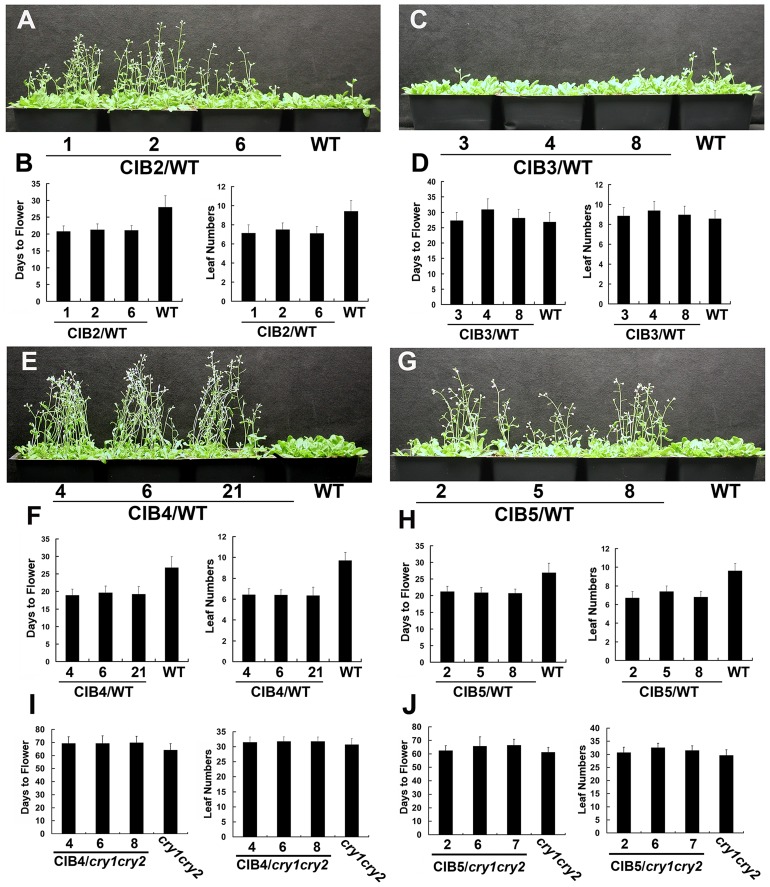
Multiple bHLH promote flower initiation in long day condition. (**A–H**) Flowering phenotype of different transgenic lines in long day. Three independent overexpress lines expressing *35S::Myc-CIB2* (A–B), *35S::Myc-CIB3* (C–D), *35S::Myc-CIB4* (E–F), *35S::Myc-CIB5* (G–H) and the WT control were grown in LD (16-h light/8-h dark) for 23 days when the pictures were taken. The quantitative flowering times measured as days to flower and the number of rosette leaves at the day floral buds became visible, and the standard deviations (n≥20) are shown. (**I–J**) Three independent overexpress lines expressing *35S::Myc-CIB4* (I) or *35S::Myc-CIB5* (J) in *cry1cry2* background and the *cry1cry2* control were grown in LD (16-h light/8-h dark). The quantitative flowering times measured as days to flower and the number of rosette leaves at the day floral buds became visible, and the standard deviations (n≥20) are shown.

Overexpression of CIB1 results in accelerated flowering in the wild-type background but not in the *cry1cry2* mutant background, demonstrating that the floral promotion activity of CIB1 is dependent on cryptochromes. However, the monogenic *cib1* mutant shows no phenotypic alterations, whereas the *cib1cib5* double mutant flowers slightly later than the wild type plants in a specific condition [14], suggesting that CIB1 acts together with additional CIB1-related proteins to promote CRY2-dependent floral initiation. In order to isolate additional CIB1-related proteins that are involved in floral initiation, we first peformed a phylogenetic analysis (Figure S1A), and found out that 6 out of 17 members of the bHLH subfamily 18, are more closely related to CIB1. We then examined their ability to interact with CRY2. 4 of the 6 CIB1-related bHLH proteins examined (CIB2-At5g48560, CIB3-At3g07340, CIB4-At1g10120, CIB5-At1g26260) interacted with CRY2 *in vitro* in a pull-down assay (Figure S1B). The two that do not interact with CRY2 were named CIL1 (At1g68920) and CIL2 (At3g23690) (CIB1 Like protein). The T-DNA insertion mutations are available for 4 of the 6 CIB1-related genes (*cib2*, *cib3*, *cib5* and *cil1*), but none of these monogenic mutants showed apparent phenotypic alterations (data not shown). Transgenic plants expressing *35S::MycCIB2*, *35S::MycCIB4*, *35S::MycCIB5*, and *35S::MycCIL1* flowered significantly earlier than the wild type parents in long day condition, while transgenic plants expressing *35S::MycCIB3* and *35S::MycCIL2* showed no obvious flowering phenotype (Figure 1A–H, Figure S2A–D). Furthermore, in contrast to transgenic plants overexpressing CIBs in the wild-type background that flowered significantly earlier than the parent, transgenic plants overexpressing CIB4 and CIB5 in the *cry1cry2* mutant background flowered at the same time as the *cry1cry2* parent in the long day condition (Figure 1 I–J), suggesting that the activity of these CIBs on floral initiation depend on CRY2 and that they also act as CRY2-signaling proteins.

### CIB proteins promote flowering redundantly by activating *FT* mRNA expression

**Figure 2:**
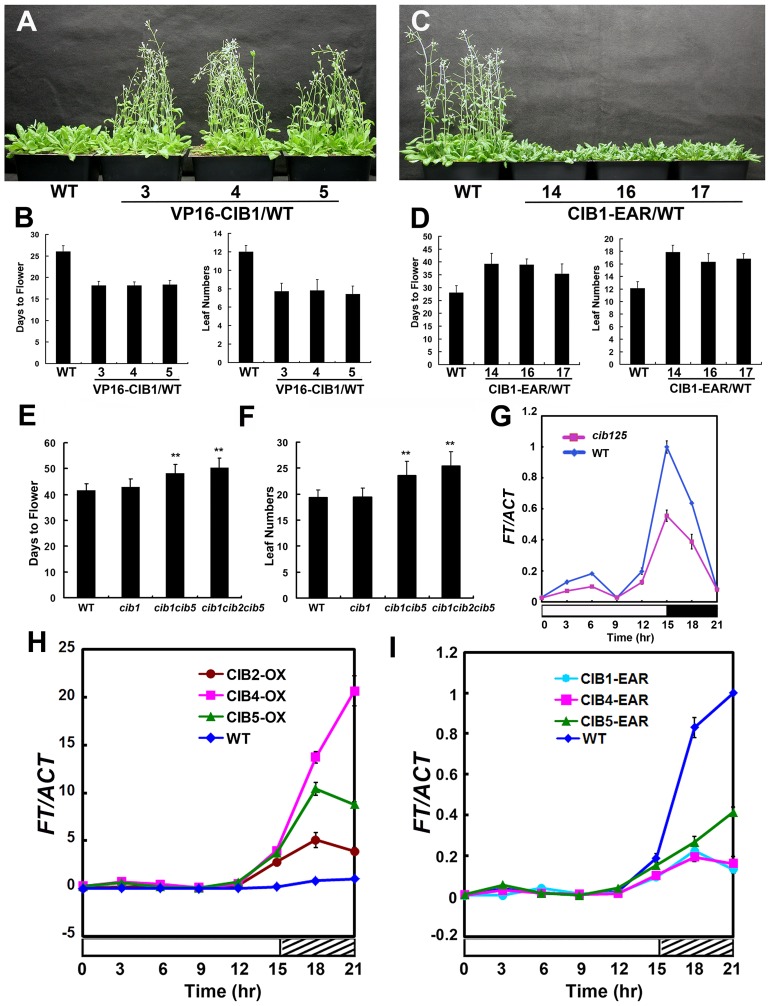
CIB proteins promote flowering redundantly by activating *FT* mRNA expression. (**A–D**) Flowering phenotype in long day. Three independent *35S::VP16-Myc-CIB1* (A–B) transgenic lines and the WT control were grown in long day for 23 days when the picture was taken. *35S::Myc-CIB1-EAR* (C–D) plants and the WT control were grown in long day for 33 days when the picture was taken. The quantitative flowering times measured as days to flower and the number of rosette leaves at the day floral buds became visible, and the standard deviations (n≥20) are shown. (**E–F**) The *cib15* double and the *cib125* triple mutant showed a mild but statistically significant delay of flowering under a photoperiodic inductive condition. Plants were grown in short-day photoperiod (9 hL/15 hD) for 20 days, transferred to long-day photoperiod (16 hL/8 hD) for 4 days, and moved back to short-day to continue grow until flowering. Days from sawing to flowering and number of rosette leaves at the time of flowering are shown with the standard deviations (n>20). (**G**) A comparison of the *FT* mRNA expression in the *cib1cib2cib5* triple mutant and the wild type. Plants were grown in short-day photoperiod (9 hL/15 hD) for 20 days and transferred to long-day photoperiod (16 hL/8 hD) for 4 days, samples were collected every 3 hr for 24 hr in the fourth day of long day at the time indicated for the qPCR analysis. (**H–I**) Quantitative PCR results showing mRNA expression of *FT* in the wild type (WT), transgenic lines expressing the *35S::Myc-CIB2, 35S::Myc-CIB4, 35S::Myc-CIB5* or *35S::Myc-CIB1-EAR, 35S::Myc-CIB4-EAR, 35S::Myc-CIB5-EAR* transgene in the wild-type background grown in long-day (16 hL/8 hD) for 6 days then moved to continue white light for one day. Samples were collected every 3 hr for 24 hr in the continuous white light. Each experiment was performed at least three times with similar results.

Considering the functional redundancy of these CIBs, we generated dominant repressor versions of CIB1, CIB4 and CIB5 using chimeric repressor silencing technology, in which CIB1, CIB4, CIB5 were fused to a 12-amino acid EAR motif, which serves as a very strong repressor domain [29]. In contrast to the Myc-CIB1 overexpression plants [14], expression of *Myc-CIB1-EAR* under the drive of *35S* promoter resulted in a marked delayed flowering phenotype (Figure 2C–D), which suggests that CIB1 functions as a transcription activator for flowering and *FT* expression. The fusion of the EAR motif does not affect the interaction between CRY2 and CIB1, as they still interact with each other in a blue light dependent manner (Figure S3). For the yeast two-hybrid assay, cells expressing both CRY2 and CIB1-EAR showed fluence rate-dependent increase of the β-gal activity after normalization for cell number. Yeast cells exposed to higher fluence rate of blue light for the same duration of irradiation exhibited higher β-gal activity (Figure S3A), suggesting a more robust interaction of CRY2 and CIB1-EAR under stronger light. As expected, cells irradiated with blue light of the same fluence rate but for a longer duration of irradiation also exhibited higher β-gal activity (Figure S3A). Then we examined the CRY2 and CIB1-EAR complex formation in plants expressing *MycCIB1-EAR*, with a coimmunoprecipitation (co-IP) assay. Seedlings were pre-treated with the proteasome inhibitor MG132 to block blue light dependent CRY2 degradation [30]. Samples were then exposed to red light, or blue light (20 µmol m−2 s−1), and subjected to co-IP analyses. CIB1-EAR was co-precipitated with CRY2 in samples irradiated with blue light but not red light (Figure S3B). Blue light stimulates the accumulation of the CRY2-CIB1-EAR complex in plant cells, like it does with the CRY2-CIB1 complex. Transgenic plants expressing *35S::Myc-VP16-CIB1*, in which CIB1 was fused to the VP16 activation motif, show an early flowering phenotype (Figure 2A–B), as observed by overexpression of CIB1, while expression of *35S::Myc-CIB4-EAR* or *35S::Myc-CIB5-EAR* also leads to a dramatic late flowering phenotype (Figure S2E–H), as observed with Myc-CIB1-EAR, which confirms that these CIBs function as transcription activators in regulating flowering.

To further test the genetic redundancy among these bHLH genes, *cib2*, *cib5*, *cil1* mutant alleles were isolated (Material Method and Figure S4). We got *cib4* (SALK_027284) seeds from ABRC, but none of the seeds have a T-DNA insertion even though the seeds were ordered twice. Plants carrying different combinations of mutations were constructed. The *cib125* triple mutant showed a statistically significant delay of flowering under the photoperiodic induction condition [8], [14] (Figure 2E–F).

Transgenic plants overexpressing CIB2, CIB4 or CIB5 exhibited elevated mRNA expression of the flowering-time gene *FT* (Figure 2H), while *cib125* triple mutant (Figure 2G) or transgenic plants overexpressing *CIB1-EAR*, *CIB4-EAR*, *CIB5-EAR* all exhibited decreased expression of *FT* (Figure 2I). We conclude that CIBs promote flowering redundantly by activating *FT* mRNA expression.

### Blue light-dependent CRY2-CIB5 interaction in plant cells

**Figure 3:**
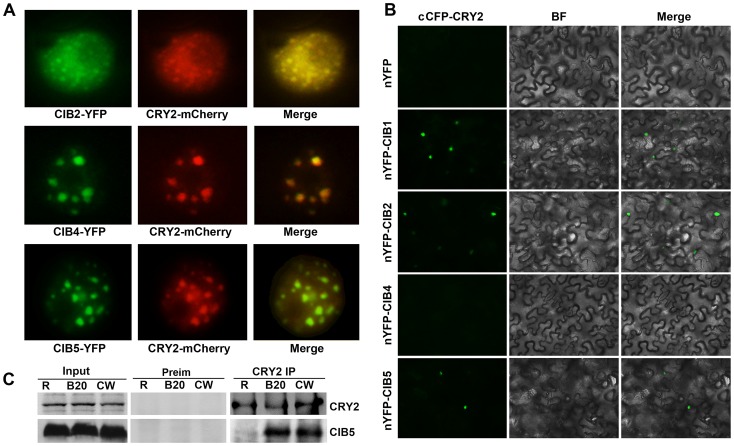
Blue light-dependent CRY2-CIB5 interaction in plant cells. (**A**) Fluorescent microscopy images showing that CIB3, CIB4 and CIB5 (Green) all co-localize with CRY2 (Red) in the nucleus. (**B**) Bimolecular fluorescence complementation assays of the *in vivo* protein interaction. Leaf epidermal cells of *N. benthamiana* were cotransformated with cCFP–CRY2 and nYFP, or nYFP-CIB1, or nYFP-CIB2, or nYFP-CIB4, or nYFP-CIB5. BF, Bright Field; Merge, overlay of the YFP and bright field images. (**C**) The co-immunoprecipitation assay showing the blue light dependent CRY2-CIB5 interaction in planta. Co-IP assays of samples prepared from 12-day-old *35S::MycCIB5* seedlings grown in continuous red light, pre-treated in MG132, then exposed to white light (W), or red light (R), or blue light (B, 20 µmol m−2 s−1, 20 min). Total proteins (Input) or IP product of anti-CRY2 antibody (CRY2-IP) or preimmune serum (Preim) were probed, in immunoblots, by the anti-CRY2 antibody (CRY2), stripped and reprobed by the anti-Myc (MycCIB1) antibody.

CIB2, CIB4, and CIB5 can all interact with CRY2 *in vitro* (Figure S1) and they are nuclear proteins. CIB2-YFP, CIB4-YFP and CIB5-YFP can all be detected in the nucleus in tobacco, and the green fluorescence of CIB2-YFP, CIB4-YFP, CIB5-YFP co-localizes with the red fluorescence of CRY2-mCherry, especially in the photobodies (Figure 3A). We examined CRY2-CIBs interaction in plant cells using the BiFC (Bimolecular fluorescence complementation) assay [31], [32], [33]. In tobacco leaf epidermal cells coexpressing the C-terminal half of CFP fused to CRY2 (cCFP–CRY2) and the N-terminal half of YFP fused to CIB1 (nYFP–CIB1), or N-terminal half of YFP fused to CIB2 (nYFP-CIB2), or nYFP-CIB5, strong YFP fluorescence was observed (Figure 3B). In contrast, no YFP signal was observed when cCFP–CRY2 and nYFP-CIB4 or no-fusion nYFP (Figure 3B), or nYFP-CIB1/2/4/5 and no-fusion cCFP, were cotransformed (data not shown). CIB2 and CIB5 but not CIB4 interact with CRY2 in planta even though all of the three interact with CRY2 *in vitro*. To further examine the *in vivo* interaction of CIB5 and CRY2, co-IP was applied. Seedlings were pre-treated with the proteasome inhibitor MG132 to block blue light dependent CRY2 degradation [30]. Samples were then exposed to red light, white light, or blue light (20 µmol m−2 s−1), and subjected to co-IP analyses. CIB5 was co-precipitated with CRY2 in samples irradiated with white light or blue light but not red light (Figure 3C). We conclude that CIB2 and CIB5 interact with CRY2 *in vivo* and at least CIB5 can undergo blue light-dependent physical interaction with CRY2 like CIB1. We did not detect the *in vivo* interaction of CRY2 and CIB4, even that CIB4 exhibited CRY2 dependent activity on floral initiation, one possibility is that CIB4 interact with CIB1 or CIB5 or other CRY2 interacting bHLH proteins to form heterodimer to regulate flowering downstream of CRY2.

### CIB4 and CIB5 associate with the chromatin regions of the *FT* gene

**Figure 4:**
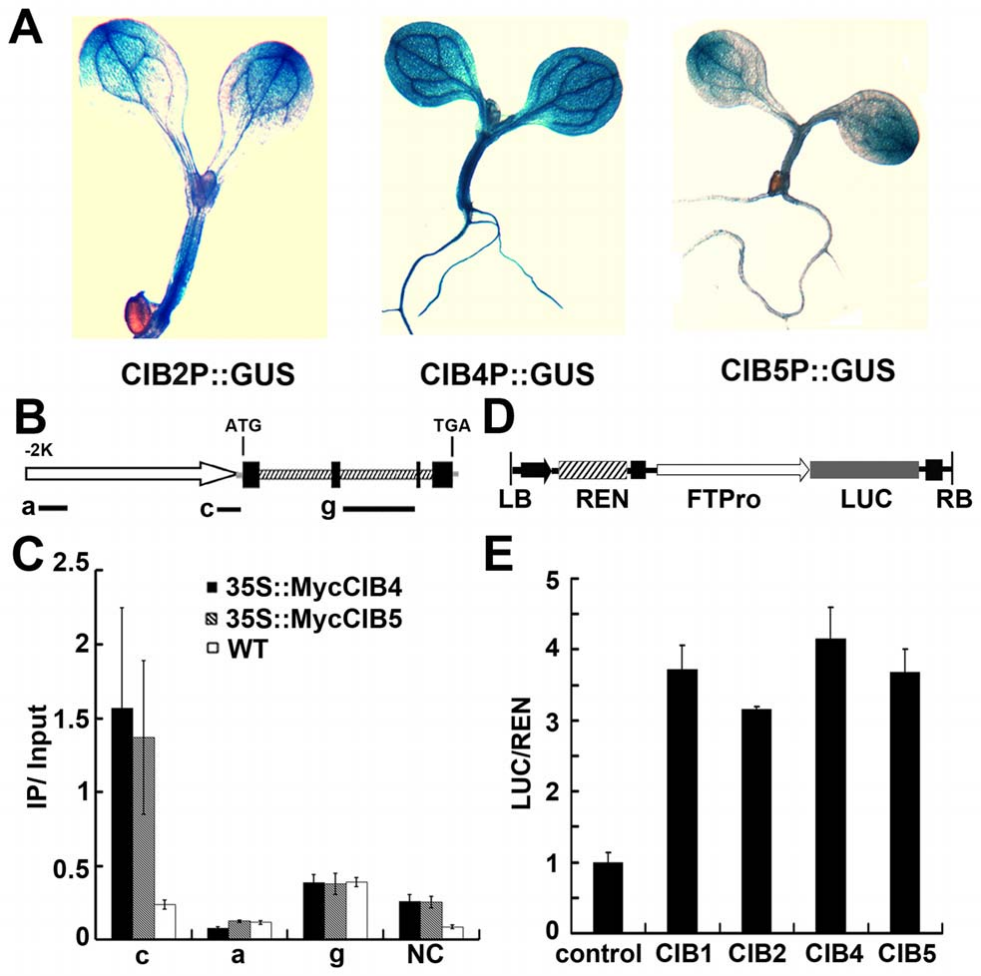
ChIP-qPCR showing interaction of CIB4 and CIB5 with chromatin regions of the *FT* gene. (**A**) GUS staining of seedlings expressing *CIB2::GUS*, *CIB4::GUS*, *CIB5::GUS* transgene. (**B**) A diagram depicting the putative promoter (arrow), 5′ UTR (grey line), exons (black boxes), introns (dashed boxes), 3′ UTR (grey line) of the *FT* gene. Black solid lines depict the DNA regions that were amplified by ChIP-PCR using the indicated primer sets. (**C**) Representative results of the ChIP-qPCR assays. Chromatin fragments (∼500 bp) were prepared from 7-day-old transgenic seedlings expressing *35S::Myc-CIB4* or *35S::Myc-CIB5*, immunoprecipitated by the anti-Myc antibody, and the precipitated DNA were qPCR-analysised using the primer pairs indicated. The IP/input ratios are shown with the standard deviations (*n*≥3). (**D**) Structure of the FT promoter–driven dual-Luc reporter gene. 35S promoter (black arrow), FT promoter (−2000 bp–0 bp) (white arrow head), REN luciferase (REN), firefly luciferase (LUC), and T-DNA (LB and RB) are indicated. (**E**) Relative reporter activity (LUC/REN) in planta with different effectors (CIB1/2/4/5) expression. Control: transiently expressed reporter only, CIB1: transiently expressed reporter and CIB1, CIB2: reporter and CIB2, CIB4: reporter and CIB4, CIB5: reporter and CIB5. Tobacco leaves were transfected with the reporter and the effector (CIB1 or CIB2 or CIB4 or CIB5); kept in white light for 3 days. The relative LUC activities normalized to the REN activity are shown (LUC/REN, *n* = 3).

CIB1 interacts with the chromatin DNA of the *FT* gene that possesses various non-canonical E-box elements but no canonical E-boxes. CIB2, CIB4, and CIB5 work redundantly with CIB1 to promote flowering by activating *FT* expression. We therefore examined whether CIB4 and CIB5 might interact with the *FT* gene as CIB1 does, using the ChIP-qPCR and Chip-PCR assay. Both the Chip-qPCR (Figure 4B–C) and Chip-PCR (Fig. S5A–C) show that *in vivo*, CIB4 and CIB5 are associated with the same chromatin region of the *FT* promoter (region c) as CIB1, which contains non-canonical E-box sequences (CAAGTG, CACCTG). Given that CRY2 control of *FT* transcription took place primarily in the vascular bundle cells [34], we also tested whether CIB2, CIB4 and CIB5 were expressed in the vascular bundle cells. Analyses of the GUS (β-glucuronidase) reporter expression in transgenic plants expressing GUS under control of the CIB2, CIB4 or CIB5 promoter demonstrated that these promoters were active in the vascular bundle cells (Figure 4A). Finally, we analyzed the transcription activity of CIBs on the *FT* promoter. A transient transcription assay in *tobacco* leaves was used. We used a dual-LUC reporter plasmid that encodes a firefly luciferase (LUC) driven by the *FT* promoter (−2000 bp to 0 bp) and a Renilla luciferase (REN) driven by the constitutive 35S promoter (Figure 4D) [14], [35]. Our result indicates that CIB1, CIB2, CIB4 or CIB5 all can promote the transcription of the *FT* promoter-LUC gene (Figure 4E). These results support our hypothesis that CIBs interact with the non-canonical E-box regulatory elements of the *FT* gene, whereas CRY2 interacts with at least CIB1 and CIB5 in response to blue light to affect *FT* transcription and floral initiation.

### CIB1 can heterodimerize with other CIBs

**Figure 5:**
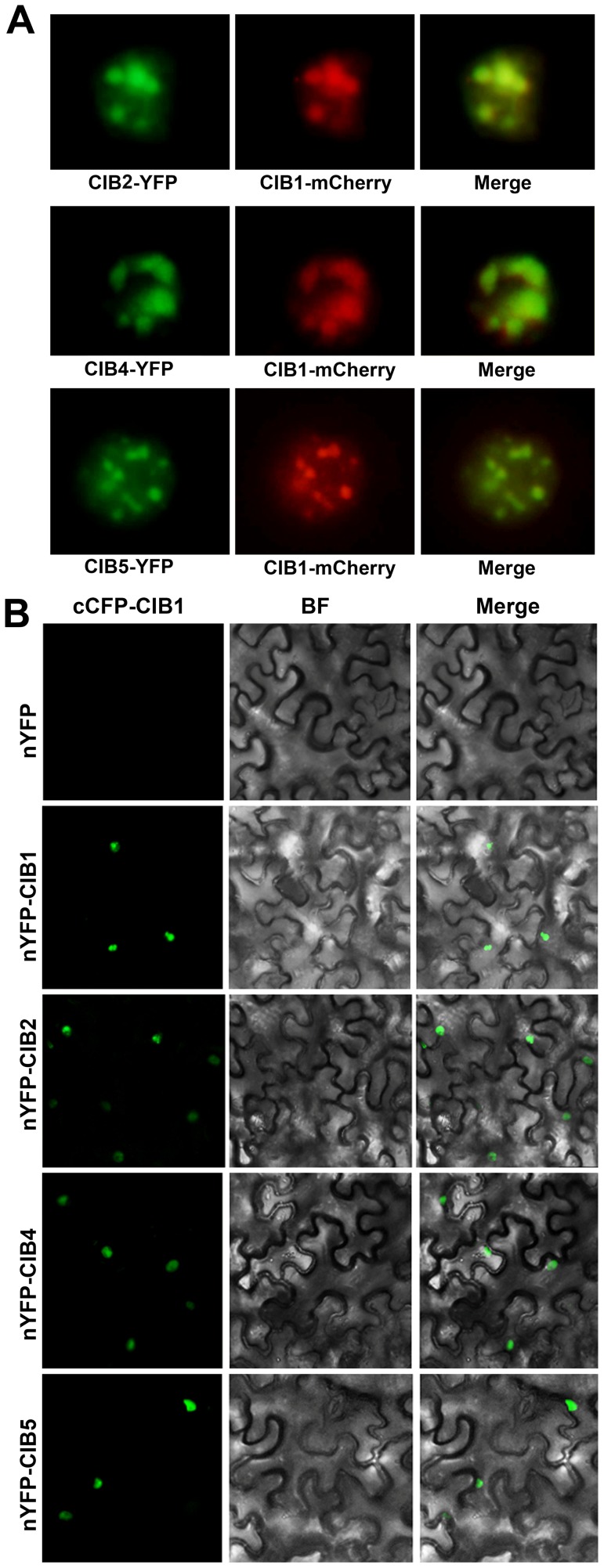
CIB1 interacts with CIBs. (**A**) Fluorescent microscopy images showing that CIB2, CIB4 and CIB5 (Green) all co-localize with CIB1 (Red) in the nucleus. (**B**) Bimolecular fluorescence complementation assays of the *in vivo* protein interaction. Leaf epidermal cells of *N. benthamiana* were cotransformated with cCFP–CIB1 and nYFP [31], or nYFP-CIB1, or nYFP-CIB2, or nYFP-CIB4, or nYFP-CIB5. BF, Bright Field; Merge, overlay of the YFP and bright field images.

The bHLH factors can form homo- or heterodimers to bind to specific DNA motifs, such as the canonical E-box (CACGTG) or the non-canonical E-box (CANNTG) [36]. CIB1 binds to the canonical E-box in vitro with a higher affinity than with other non-canonical E-box elements. However, CIB1 binds to the chromatin region of the *FT* promoter *in vivo*, which only contains the non-canonical E-box but not the canonical E-box [14]. We hypothesized that CIB1 works redundantly with other CIB1-related bHLH proteins, and different CIB proteins may heterodimerize to interact with the non-canonical E-box DNA *in vivo*. We already showed that CIB proteins promote flowering redundantly by activating *FT* mRNA expression, and CRY2 interacts with at least CIB1 and CIB5 in response to blue light to affect *FT* transcription and floral initiation. To further test whether CIB1 can form heterodimers with CIB2, CIB4 and CIB5, we first checked the co-localization of CIB1 and CIB2, CIB4, CIB5. The green fluorescence of CIB2-YFP, CIB4-YFP, CIB5-YFP co-localizes with the red fluorescence of CIB1-mCherry (Figure 5A). We used the bimolecular fluorescence complementation assay to check the interaction of CIB1 with CIBs [31], [32], [33]. In tobacco leaf epidermal cells coexpressing the C-terminal half of CFP fused to CIB1 (cCFP–CIB1) and the N-terminal half of YFP fused to CIB1 (nYFP–CIB1), or N-terminal half of YFP fused to CIB2 (nYFP-CIB2), or nYFP-CIB4 or nYFP-CIB5, strong YFP fluorescence was observed (Figure 5B). In contrast, no YFP signal was observed when cCFP–CIB1 and no-fusion nYFP (Figure 5B), or nYFP-CIB1/2/4/5 and no-fusion cCFP, were cotransformed (data not shown). These indicate that CIB1 can form heterodimer with CIB2, CIB4 and CIB5 in planta.

### CIB heterodimers bind to the non-canonical E-box sequence of the *FT* promoter *in vitro*

**Figure 6:**
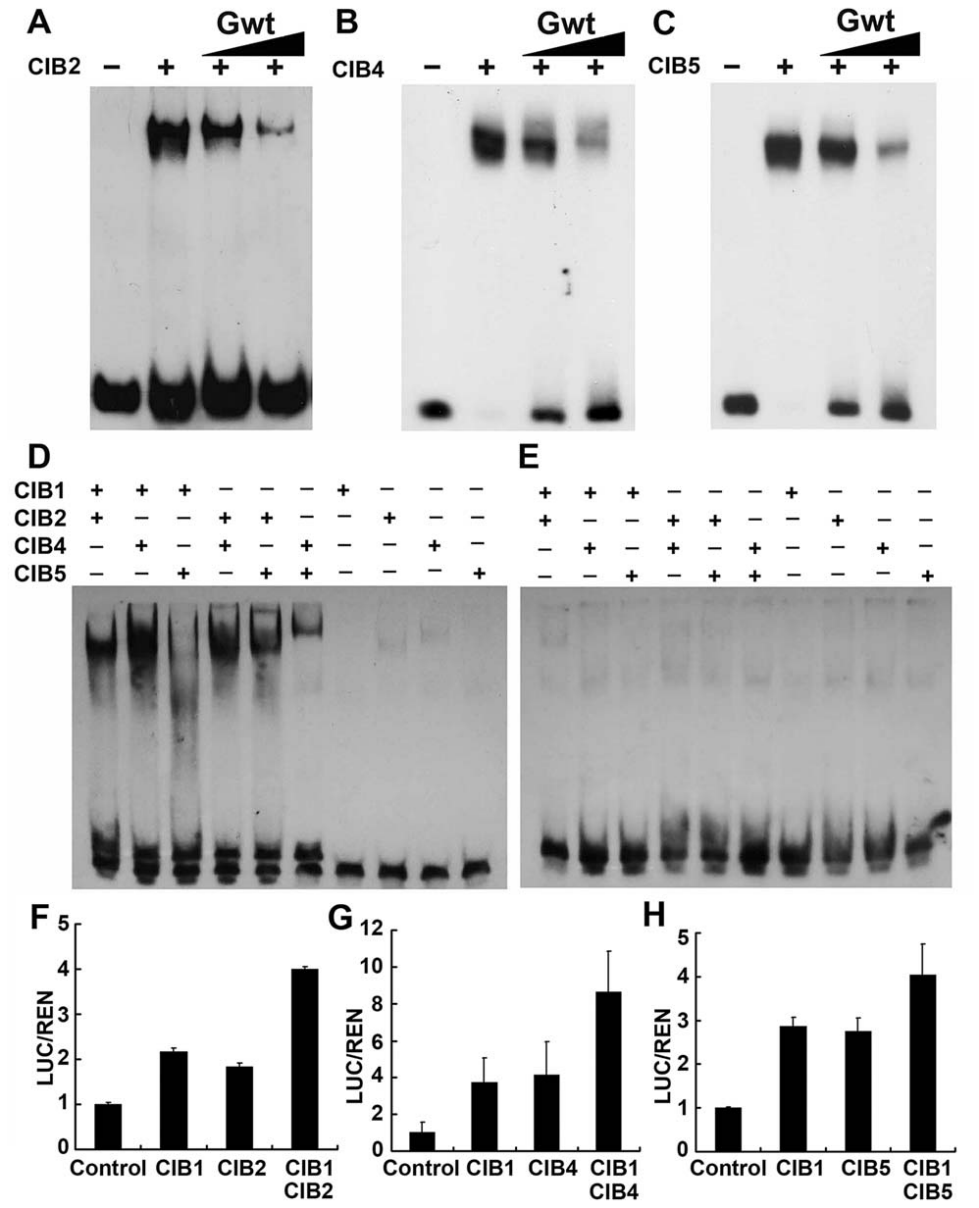
CIB heterodimers bind to the non-canonical E-box sequence of the *FT* promoter. (**A–C**) Competitive electrophoretic mobility shift assay (EMSA) showing binding of CIB2 (A), CIB4 (B), and CIB5 (C) to the G-box DNA (canonical E-box) *in vitro*. Relative amounts of the un-labeled competitive oligonucleotide containing the G-box sequence used in the reactions are indicated on the top. (**D–E**) An EMSA experiment showing association of the *CIB* heterodimers, but not monomers, with the non-canonical E-box DNA of the *FT* promoter (region c in Figure 4B). The indicated CIB proteins were expressed and purified from *E. coli*, and incubated with the labeled oligonucleotide containing the E-box DNA of the *FT* promoter (D) or the same sequence except that the E box was replaced with AAAAAA sequence (E) (**F–H**) Transient assays show CIBs (CIB1/2/4/5) activation of the *FTpro::LUC* reporter gene. (F) Control: transiently expressed reporter only, CIB1: reporter and CIB1, CIB2: reporter and CIB2, CIB1 CIB2: reporter, CIB1 and CIB2 together. (G) CIB4: reporter and CIB4, CIB1 CIB4: reporter, CIB1 and CIB4. (H) CIB5: reporter and CIB5, CIB1CIB5: reporter, CIB1 and CIB5. Tobacco leaves were transfected with the reporter and the effectors; kept in white light for 3 days. The relative LUC activities normalized to the REN activity are shown (LUC/REN, *n* = 3). Error bars indicate SD of three biological repeats.

CIB1 binds to the canonical E-box (G-box) with highest binding affinity *in vitro*, although it shows similar binding affinity to both canonical and non-canonical E-boxes *in vivo*. The specificity of the interaction between CIB2, CIB4, CIB5 and the canonical E-box were verified using electrophoretic mobility shift assays (EMSAs). The result confirmed that they all bind specifically to the canonical E-box *in vitro* (Fgiure 6A–C), and a single-nucleotide mutation within the canonical E-box sequence significantly reduced the ability of the mutant DNA to interact with CIB2, CIB4, and CIB5 (Figure S6A–C). CIB1, CIB2, CIB4, and CIB5 all bind to the canonical E-box *in vitro* with a higher affinity than their interaction with other non-canonical E-box DNA sequence. However, CIB1, CIB4, and CIB5 all associated with the *FT* promoter *in vivo*, which lacks a canonical E-box but contains several non-canonical E-boxes (CAAGTG, CACCTG). We hypothesized that different CIBs may form heterodimers to interact with the non-canonical E-box DNA *in vivo*. We already showed that CIB1 can heterodimerize with CIBs. To further test this hypothesis, we did an *in vitro* EMSA assay by using different combination of the CIB proteins. As we expected, the CIB1–CIB3, CIB1–CIB4, CIB2–CIB4, CIB2–CIB5, and CIB4–CIB5 heterodimers all bind to the non-canonical E-box sequence of the *FT* promoter (−334 to −311 bp, sequence:AGTGGCTACCAAGTGGGAGATATA), while CIB1–CIB5 does not (Figure 6D). The combination of CIB1 with CIB2 or CIB4 did not significantly change the binding affinity of CIB1 to canonical E-box since the binding affinity of CIB1 to canonical E-box is already very high (Figure S6D–E). To further test our hypothesis, the transient dual-LUC assay was employed again. The *FTpro-LUC* (Figure 4E) reporter was infiltrated into tobacco leaves together with Agrobacteria cells harboring one CIB (either CIB1, CIB2, CIB4 or CIB5) or with half the amount of CIB1 plus half the amount of CIB2, or CIB4 or CIB5. The expression level of *FT promoter-LUC* is about two times higher when CIB1 was combined with CIB2 or CIB4 than when only one CIB protein was infiltrated, even though the same amount of Agrobacteria cells were infiltrated (Figure 6 F–G). The transcription of the reporter is increased about 30% percent with the combination of CIB1 and CIB5 (Figure 6H). All these results indicate that although CIB proteins have the highest affinity for the canonical E-box *in vitro*, the heterodimers of different CIBs proteins bind the non-canonical E-box elements both *in vivo* and *in vitro*.

### CIBs proteins are regulated exclusively by blue light

**Figure 7:**
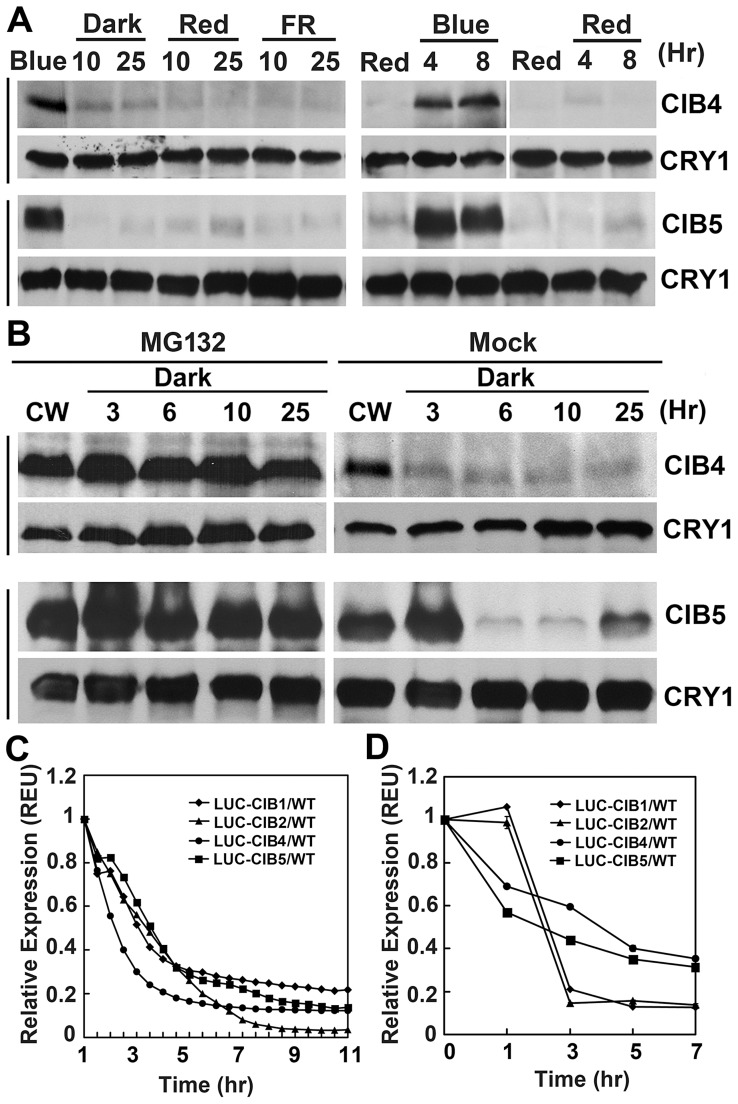
Immunoblots and luciferase assays showing light regulation of CIBs protein expression. (**A**) Transgenic plants expressing the *35S::Myc-CIB4* and *35S::Myc-CIB5* transgenes were grown in long day for 3 weeks, treated with blue light (Blue) for 16 hr, and transferred to dark (Dark), red light (Red, 20 µmol m−2 s−1), or far red light (FR, 5 µmol m−2 s−1) for the indicated time (Left). Alternatively, the 3-week-old plants were first treated with red light for 16 hr (Red), and transferred to blue light (Blue, 35 µmol m−2 s−1) or kept in red light (Red, 20 µmol m−2 s−1) for the indicated time. (**B**) Immunoblot showing the inhibition of CIB4 and CIB5 degradation in darkness by the proteasome inhibitor MG132. Plants expressing the *35S::Myc-CIB4* or *35S::Myc-CIB5* transgenes were grown in continuous white light (CW) for 3 weeks, leaves were excised and incubated with MG132 (50 µmol/L) or mock solution (0.1% DMSO) in darkness for the indicated time. (**C–D**) A luciferase assay showing decreased levels of LUC-CIB1, LUC-CIB2, LUC-CIB4, and LUC-CIB5 fusion proteins in the absence of blue light. Transgenic *Arabidopsis* seedlings expressing the indicated LUC-fusion CIB proteins were grown in continuous blue light for 7 days, and transferred to dark (C) or red light (D) for the indicated time. The luciferase activity was measured by a CCD camera (C) or by a luminometer (D). For (C)), the bioluminescence/20 seedlings were measured by a CCD camera and shown after background subtraction. For (D), the relative levels of LUC activity (REU) was calculated by the formula [LUC^Red^/mg^Red^]/[LUC^Blue^/mg^Blue^]. LUC^Blue^ and LUC^Red^: luciferase activity of dark- or blue light-treated samples, mg^Red^ and mg^Blue^: total proteins (mg) of dark- or blue light-treated samples.

As we know, most of the proteins involved in light signal transduction are light regulated, such as CRY2 protein which gets degraded under blue light [4], [37], PHYA protein undergoes rapid degradation in red light [38], and PIFs get degraded in the presence of red light [39]. Consistent with the hypothesis that CIBs act as CRY2-signaling proteins, they are also blue light regulated. Similar to CIB1 [40], the expression of CIB2, CIB4, and CIB5 proteins are regulated by blue light in a wavelength-specific manner. To study the regulation of these proteins, we used transgenic plants expressing either the Myc-tagged CIB4 or Myc-tagged CIB5 fusion protein, which are controlled by the constitutive 35S promoter (*35S:Myc-CIB4, 35S:Myc-CIB5*), and the luciferase tagged CIB1, CIB2, CIB4, or CIB5 fusion proteins which are under the control of the 35S constitutive promoter (*35S:LUC-CIB1, 35S:LUC-CIB2, 35S:LUC-CIB4, 35S:LUC-CIB5*). For unknown reasons, neither of the Myc-CIB2 or Flag-CIB2 fusion proteins was detected by the immunoblots, although they all showed early flowering phenotype and overexpressed mRNA. The immunoblot experiments showed that CIB4 and CIB5 proteins were barely detectable in plants grown in the dark or red light, but they started to accumulate soon after plants were exposed to blue light (Figure 7A, Figure S7). While abundant CIB4 and CIB5 proteins were detected in plants exposed to blue light, the CIB4, CIB5 protein level decreased after plants were transferred from blue light to dark, red light, or far-red light (Figure 7A, Figure S7). Results of the luciferase activity analysis corroborated with the immunoblot analysis. LUC-CIB1, LUC-CIB2, LUC-CIB4, LUC-CIB5 fusion proteins all get degraded after the plants were moved from blue light to dark condition in two different luciferase assays, the *planta* bioluminescence assay (Figure 7C) and the luciferase assay of plant extracts (Figure 7D). Treatment of Myc-CIB4 and Myc-CIB5 OX seedlings with the 26S proteasome inhibitor MG132 prevented the decline of CIB4 and CIB5 proteins abundance in the absence of blue light (Figure 7B). EAR fusion does not affect protein degradation since Myc-CIB1-EAR fusion protein also gets degraded without blue light but is accumulated in the blue light condition (Figure S7). These results demonstrate that like CIB1, in the absence of blue light, CIB2, CIB4 and CIB5 are also degraded by the 26S proteasome, and that blue light suppresses their degradation.

## Discussion

We investigated the function and biochemical mechanisms of 3 CIB1-related proteins, CIB2, CIB4, and CIB5 in this study. We showed that CIB1, CIB2, CIB4, and CIB5 function redundantly to activate the transcription of *FT* and that they are positive regulators of CRY2 mediated flowering. CIB1 and the CIB1 related bHLHs can form heterodimers and some of those heterodimers have higher binding affinity to the non-canonical E-box, which explains why CIB1 and other CIBs binds to the canonical E-box (CACGTG, G-box) *in vitro* with a higher affinity than to the non-canonical E-box elements (CANNTG), while they all associate with the *FT* chromatin which only contains non-canonical E-boxes. To our knowledge, this is the first evidence in plants that heterodimerization of distinct bHLH proteins can affect the specificity of the elements bound by bHLH proteins. Consistent with our hypothesis that CIBs are specifically involved in CRY2 signaling, the expression of CIBs proteins is regulated specifically by blue light. Our study demonstrates that CIBs function redundantly in regulating CRY2-mediated flowering, and more importantly, different CIBs genes form heterodimers to interact with the non-canonical E-box DNA *in vivo*.

### CIBs act redundantly to promote flowering

The bHLH proteins are one of the largest transcription factor families in eukaryotes, and there are about 170 bHLH proteins in *Arabidopsis*. bHLH transcription factors can form homo- or heterodimers through their HLH domain, and they bind to canonical E-box (G-box) or non-canonical E-box through the basic domain [36]. In *Arabidopsis*, phytochromes interact with several bHLH transcription factors, known as PIF proteins, in a light dependent manner to modulate phytochrome function and regulation [15]–[21]. Cryptochromes regulate gene expression by modulating activities of the circadian clock [41], and interact directly with transcription factors, such as CIB1 to regulate gene transcription [14]. We showed that several CIB1 related bHLH genes, *CIB2*, *CIB4*, *CIB5*, and *CIL1* all can promote flowering initiation in the long day condition. Expression of the dominant repressor version of CIB1, CIB4 or CIB5 resulted in a marked delayed flowering phenotype. Impairment of three of these bHLH genes in the *cib125* triple mutant caused a statistically significant delay of flowering under the photoperiodic induction condition [8], [14], demonstrating that these genes are essential for the CRY2 mediated flowering in the wild type plants. Nevertheless, there may still be other CIB related proteins involved, such as CIL1, since overexpression of CIL1 also results in an early flowering phenotype. There are also other bHLH members in the same clade with CIB1, such as BEEs [42].

CIBs belong to the subfamily 18 of bHLH proteins. The subfamily 18 contains 17 members, including *BEE1* (*BR enhanced expression*), *BEE2*, and *BEE3* [36], [42]. The mRNA expression of the three *BEE* genes and some other members of this subfamily are regulated by brassinosteroids [42]. Genetic analysis demonstrates that the three BEE proteins are functionally redundant positive regulators of brassinosteroids signaling [42]. Among other members of this subfamily, CESTA, is a positive regulator of brassinosteroids biosynthesis [43] while BIGPETALp (BPEp) affects *Arabidopsis thaliana* petal growth by influencing cell expansion [44]. Very recently, ACEs/CIB5 was reported to be involved in regulating cell elongation, where it was shown that PRE1 (a HLH protein that regulate growth downstream of a wild range of signals) [45], IBH1 (HLH factor that inhibit cell elongation) [45], [46], and the ACEs constitute a triantagonistic bHLH system, that competitively regulates cell elongation. ACEs/CIB5 activates the enzyme genes for cell elongation directly, while IBH1 negatively regulates cell elongation by interacting with ACEs to interfere with DNA binding. PER1 interacts with IBH1 so that IBH1 can not affect ACEs [45]. ACE1 is actually our CIL1, ACE2 is our CIB4, while ACE3 is our CIL2, and we also observed a mild hypocotyle phenotype of these overexpression lines (data not shown). Zhiyong Wang's group also showed that PRE1, IBH1 and HBI1 work together and formed an antagonistic switch to regulate cell elongation under the control of multiple external and endogenous signals [47]. HBI1 is also a member of subfamily 18. CIB4, CIB5 and CIL1 promote flowering initiation together with CIB1 and CIB2, while they are also involved in regulating cell elongation, so these bHLH transcription factors may play different roles in different signal transduction pathways, and regulate different target genes.

### CIB heterodimers bind to the non-canonical E-box sequence of the *FT* promoter *in vivo*

bHLH proteins are well known to dimerize, they can form both homodimers and heterodimers [36]. bHLH transcription factors can form a heterodimer with HLH proteins, HLH proteins interact with bHLH proteins to interfere with the DNA binding of the bHLH protein, for example, PRE1 and IBH1 can dimerize with ACEs or HBI1 to regulate the cell elongation [45], [47]. It was reported previously that mouse cryptochromes physically interact with two bHLH proteins, CLOCK and BMAL, to suppress their activity of the E-box–dependent transcription. CLOCK and BMAL form heterodimers to regulate transcription [48], [49]. In plants, the bHLH transcription factor INDEHISCENT (IND) and SPATULA (SPT) can interact with each other to regulate tissue patterning in *Arabidopsis* [50]. The bHLH protein LONG HYPOCOTYL IN FAR-RED 1 (HFR1), plays a role in photomorphogenesis by forming non-DNA binding heterodimers with PIFs [51], [52]. Recently, it was reported that the HLH protein KIDARI (KDR) can dimerize with HFR, so that HFR cannot interact with PIF4 [53]. The HLH protein PAR1 can also interact with PIF4 to inhibits PIF4 mediated gene activation, while the HLH protein PRE1 interact with PAR1 to activate PIF4 [54]. These revealed that the PIF4 activity is regulated through a double layer of competitive inhibition of HFR1 and KDR [53] or PAR1 and PRE1 [54]. PIF3 and PIF4 can also form heterodimers, and the heterodimers are still capable of recognizing the G-box motif in a sequence-specific manner, the same as the PIF3 or PIF4 homodimers [36]. In *C. elegans*, some bHLH proteins can form heterodimers, and none of those participate in herodimeric interactions exhibit significant sequence-specific DNA binding on their own, they exhibit sequence-specific DNA-binding only when they form heterodimers [55]. Here we reported that CIB1 can dimerize with CIB2, CIB4 and CIB5 *in vivo* (Figure 5). CIB1, CIB2, CIB4, CIB5 all bind to the canonical E-box (G-box) with a much higher affinity than with other non-canonical E-box DNA sequence *in vitro* [14] (Figure 6A, Figure S5). However, CIB1, CIB4, CIB5 all associated with the *FT* promoter *in vivo*, which contains only non-canonical E-boxes but not canonical E-boxes [14] (Figure 4). The CIB1–CIB2, CIB1–CIB4, CIB2–CIB4, CIB2–CIB5, and CIB4–CIB5 heterodimers all bind to the non-canonical E-box sequence of the *FT* promoter *in vitro* (Fgiure 6D). Furthermore, expression of CIB1 and CIB2 or CIB1 and CIB4 together promotes the expression of the *FT* promoter-LUC to a much higher level compared with expression only one of them (Figure 6 F, G). Although CIB proteins have the highest affinity to the canonical E-box (G-box) *in vitro*, the heterodimers of different CIB proteins bind non-canonical E-box elements both *in vivo* and *in vitro*. We show direct evidence here that a bHLH protein can dimerize with more than one partner and to form heterodimers, and furthermore, that heterodimerization can modulate the DNA binding affinity of those bHLH transcription factors. Heterodimerization may be very important for the specificity of bHLH proteins.

### CIBs proteins are regulated specifically by blue light

Most of the proteins that are involved in light signaling are light regulated. For example, the photoreceptor CRY2 protein gets degraded under blue light [37], [56], [57] while phyA undergoes rapid degradation in red light [38], ZTL is stabilized under blue light [26]. Phytochromes interact with PIFs in response to light and induce rapid phosphorylation, poly-ubiquitylation and degradation of PIFs through the ubiquitin/26S proteasome pathway to promote photomorphogenesis [39]. Consistent with our hypothesis that CIBs are specifically involved in blue light signaling, we discovered that the protein expression of CIBs is regulated specifically by blue light. CIB1, CIB2, CIB4, and CIB5 proteins are degraded in the absence of blue light, via the ubiquitin/26S proteasome pathway, in the dark, red, and FR light (Figure 7, Figure S7) [40]. The degradation of CIBs are suppressed in blue light, resulting in the accumulation of CIBs in blue light, CIBs are unique compared to other light-signaling proteins that showed light-regulated protein turnover.

## Materials and Methods

### Plant materials

Except where indicated, the Columbia ecotype of *Arabidopsis* was used. The *cry1cry2, cib1, cib5, cib1cib5* mutants have been previously described. The *cib2* T-DNA insertion mutant (SALK_055827) was obtained from ABRC (http://www.arabidopsis.org/index.jsp). Transgenic *Arabidopsis* lines were prepared by floral dip transformation method [58], [59]. Phenotypes of transgenic plants were verified in at least 3 independent transgenic lines. The binary plasmids encoding the *35S:Myc-CIB2, 35S:Myc-CIB3*, *35S:Myc-CIB4, 35S:Myc-CIB5, 35S:Myc-CIL1, 35S:Myc-CIL2, 35S:Myc-CIB1EAR, 35S:Myc-CIB4EAR, 35S:Myc-CIB5EAR, 35S:Myc-VP16CIB1, 35S:LUC-CIB1*, *35S:LUC-CIB2*, *35S:LUC-CIB4, 35S:LUC-CIB5, CIB2P:GUS, CIB4P:GUS, CIB5P:GUS, 35S:CIB2-YFP, 35S:CIB4-YFP, 35S:CIB5-YFP, 35S:CRY2-mCherry, 35S:CIB1-mCherry* were prepared by conventional and/or GATEWAY methods. *CIB2P*, *CIB4P* and *CIB5P* represent the *CIB2* promoter (−2150 nt to −1 nt), *CIB4* promoter (−2592 nt to −1 nt) or *CIB5* promoter (−1752 nt to −1 nt), respectively. The *cib1cib3cib5* triple mutant was prepared by genetic crosses.

### The *in vitro* pull-down

The *in vitro* pull-down protein-protein interaction assay was modified from that described previously [14]. CRY2 protein expressed and purified from insect cells was incubated with the S35 labled CIB proteins prepared by the *in vitro* transcription/translation reactions (TnT, Promega). Ni-affinity beads were used to pull down the protein complexes.

### Co-localization and BiFC assay

The BiFC assay was based on that described previously with slight modifications [14], [33], CRY2 or CIB1 and CIBs were fused to N-terminus of YFP or C-terminus of CFP, transformed to Agrobacterium strain GV3101 containing pSoup-P19 plasmid that encodes the suppressor of gene silencing [35]. Overnight cultures of Agrobacteria were collected by centrifugation, re-suspended in MES buffer to 0.8 OD600, mixed, and incubated at room temperature for 2 hr before infiltration. Agrobacteria suspension in a 2 ml syringe (without the metal needle) was carefully press-infiltrated manually onto healthy leaves of 3-week-old *Nicotiana benthamiana*. Plants were left under continuous white light for 3 day after infiltration.

### Luciferase assays

*In planta* bioluminescence was analyzed by a cool CCD camera as previously described [60]. To compare the level of expression of LUC-fusion proteins *in planta*, plants were sprayed with luciferin solution (1 mM luciferin and 0.01% Triton X-100), image captured 5 min later by a CCD camera [61], and analyzed using the Image J software (http://rsb.info.nih.gov/ij/). The luciferase activity of plant extract was analyzed by a luminometer (Promega 20/20), using commercial LUC reaction reagents according to the manufacturer's instruction (Promega).

### Immunoblot

Immunoblot is as described previously [14], [30], [62]. Our attempts to prepare the anti-CIB1, anti-CIB2, anti-CIB4 and anti-CIB5 antibodies resulted in antisera that recognized CIB1, CIB2, CIB4, CIB5 proteins expressed in *E.coli*, but not plant proteins. For immunoblots, a mouse monoclonal anti-Myc antibody 4A6 (Millipore, #05-724, 1∶4000 dilution for immunoblot and 1∶100 for immunostain) was used to detect Myc-CIB1, Myc-CIB1-EAR, Myc-CIB4, and Myc-CIB5 fusion proteins.

### co-immunoprecipitation

co-immunoprecipitation (co-IP) is as described previously [14]. For co-IP, 12-day-old 35S::Myc-CIB5 seedlings grown in continuous red light were used, tissues were excised and incubated in MG132 for 3 hour before exposed to white light (W), blue light (B) or red light (R) for 20 minute, grounded in liquid nitrogen, homogenized in Binding Buffer (20 mM HEPES [pH 7.5], 40 mM KCl, 1 mM EDTA, 1% Triton X-100, 1 mM PMSF), and centrifuged at 16,000 g for 15 min. 1 ml supernatant was mixed with 25 µl anti-CRY2-IgG-coupled protein-A Sepharose, incubated at 4°C for 30 min. 5 µl anti-CRY2 antiserum was incubated with 20 µl protein-A Sepharose beads in a 100 µl binding, at 4°C for 2 hour, and used soon after. The mixture was transferred to a spin cup (Pierce), washed (ca. 20 sec each) 5 times with Washing Buffer (20 mM HEPES [pH 7.5], 40 mM KCl, 1 mM EDTA, 0.1% Triton X-100). The bound proteins were eluted from the affinity beads with 4× SDS-PAGE sample buffer, and analyzed by immunoblot.

### EMSA assay

The EMSA assay was as described [14]. CIB1, CIB3,CIB4 and CIB5 were expressed in *E. coli* using the pCOLD-TF expression system according to the manufacturer's instructions (Takara Bio Inc. Cat#3365). His-TF-CIB1, His-TF-CIB3, His-TF-CIB4 and His-TF-CIB5 fusion proteins were purified using Ni-affinity chromatography. The synthetic oligonucleotides (Table S1) were PCR amplified, and labeled with DIG (digoxigenin) by terminal transferase according to the manufacturer's instruction (DIG Gel Shift Kit, Roche). Total 100 ng protein was added in each binding reaction, when two CIBs protein were added, each was 50 ng.

### qPCR and GUS assays

Total RNAs were isolated using the Illustra RNAspin Mini kit (GE healthcare). cDNA was synthesized from 1 µg total RNA using SuperScript first-strand cDNA synthesis system (Invitrogen). Platinum SYBR Green qPCR Supermix-UDG (Invitrogen) or SYBR Premix Ex Tag (Takara) was used for qPCR reaction, using the MX3000 System (Stratagene). The level of *ACTIN* mRNA expression (At3g18780, Table S1) was used as the internal control. The expression of GUS (beta-glucuronidase) was analyzed as described [63].

## Supporting Information

Figure S1 Multiple bHLH proteins interact with CRY2. (A) Neighbor-joining phylogenetic analysis (MEGA4) showing phylogenetic relationship of CIB1 and other CIB family members. The bootstrap values (1000 replicates) are indicated. The scale bar indicates substitution per site. (B) *In vitro* pull-down experiment showing CIB-CRY2 interactions, and the lack of CIL-CRY2 interactions. CRY2 protein expressed and purified from insect cells was incubated with the S35 labled CIB proteins prepared by the *in vitro* transcription/translation reactions (TnT, Promega).
(TIF)

Figure S2 CIB4, CIB5 and CIL1 but not CIL2 promote flowering. Flowering phenotype in long day. Plants expressing the *35S::Myc-CIL1* (CIL1/WT) or *35S::Myc-CIL2* (CIL2/WT) in the wild-type background (A–D) were grown in long-day photoperiod (16 hL/8 hD) for 23 days when the pictures were taken. CIB4-EAR (E–F), CIB5-EAR (G–H) plants and the WT control were grown in long day for 33 days when the pictures were taken. The quantitative flowering times measured as days to flower and the number of rosette leaves at the day floral buds became visible, and the standard deviations (n≥20) are shown.
(TIF)

Figure S3 CIB1-EAR interacts with CRY2 in a blue light dependent manner. (A) β-gal assays of yeast cells expressing CIB1-EAR and CRY2 proteins irradiated with red light (R18, 18 µmol m−2 s−1) or blue light (B16 to B50, 16 to 50 µmol m^−2^ s^−1^) for the durations indicated. (B) co-IP experiment showing the blue light-dependent CRY2-CIB1EAR complex *in vivo*. 7-day-old seedlings expressing *35S::Myc-CIB1-EAR* was grown in red light, pre-treated in MG132, and transferred to blue light (20 µmol m−2 s−1)(B20), and the IP products of the anti-CRY2 antibody were analyzed by immunoblot probed with anti-CRY2 (CRY2) or anti-Myc (CIB1) antibodies.
(TIF)

Figure S4 Analysis of the *cib1cib2cib5* triple mutant. (A) Schematic illustrating the genomic structures of CIB1, CIB2, and CIB5 and the locations of the T-DNA insertions. Black boxes and striped boxes indicate exons and introns, respectively. T-DNA insertion sites are indicated by triangles. (B) RT-PCR analysis of *CIB1*, *CIB2*, *CIB5* and *Actin2* transcript abundance in wild-type (WT), *cib1*, *cib15* and *cib125* triple mutant lines. *Actin2* was used as an internal control. Data shown represent one of three independent assays that gave the same results.
(TIF)

Figure S5 ChIP-PCR showing interaction of CIB4 and CIB5 with chromatin regions of the *FT* gene. (A) A diagram depicting the putative promoter (arrow), 5′ UTR (grey line), exons (black boxes), introns (dashed boxes), 3′ UTR (grey line) of the *FT* gene. Black solid lines depict the DNA regions that were amplified by ChIP-PCR using the indicated primer sets. (B) Representative results of the ChIP-PCR assays. Chromatin fragments (∼500 bp) were prepared from 7-day-old transgenic seedlings expressing *35S::Myc-CIB4* or *35S::Myc-CIB5*, immunoprecipitated by the anti-Myc antibody, and the precipitated DNA PCR-amplified using the primer pairs indicated. Input: PCR reactions using the samples before immunoprecipitation. (C) ChIP-PCR results for the primer pairs that were repeated at least three times were quantified by normalization of the Myc-IP signal with the corresponding input signal (IP/input). The standard deviations (*n*≥3) are shown.
(TIF)

Figure S6 CIB2, CIB4, CIB5 bind to G-box specificly in vitro. (A–C) A competitive EMSA showing interaction of CIB2, CIB4, CIB5 with the DIG-labeled G-box (canonical E-box), and lack of a strong competition by the mutant G-box (Gm3: CAAGTG). Black wedges represent increasing amount of competitors (12.5×, 25×, 50× in molar excess). (D) An EMSA experiment showing association of the CIB1CIB2 *or* CIB1CIB4 heterodimers, and also CIB1 monomer, with the G-box DNA (CACGTG, canonical E-box). The indicated CIB proteins were expressed and purified from *E. coli*, and incubated with the labeled oligonucleotide containing the G-box (canonical E-box). (E) A semi-quantitative analysis of DNA binding of the EMSA shown in (D). The film shown in (D) were scanned, and analyzed by Image J software.
(TIF)

Figure S7 CIB4, CIB5 and CIB1-EAR are degraded in the absence of blue light. (A–D) Immunoblot experiments showing the light regulation of CIB4, CIB5 protein expression in transgenic plants expressing the *35S::Myc-CIB4* or *35S::Myc-CIB5* transgene. Samples were fractionated by 10% SDS-PAGE, blotted, and probed by the anti-Myc antibody, stripped and re-probed with the anti-CRY1 antibody to indicate relative loading of the samples. In the first experiment (A, C), 3-week-old long day-grown (16 hL/8 hD) plants were transferred to dark for 16 hr, and then transferred to blue light (35 µmol m^−2^ s^−1^) for the indicated time before sample collection. In the second experiment (B, D), 3-week-old long day-grown plants were transferred to continuous blue light (Blue, 35 µmol m^−2^ s^−1^) for 16 hr, and then transferred to dark for the indicated time. (E–F) Immunoblot experiments showing the light regulation of not only CIB1 but also CIB1-EAR protein expression in transgenic plants expressing the *35S::Myc-CIB1* or *35S::Myc-CIB1-EAR* transgene. (E) 3-week-old long day-grown (16 hL/8 hD) plants were transferred to red light (20 µmol m^−2^ s^−1^) for 16 hr, and then transferred to blue light (35 µmol m^−2^ s^−1^) for the indicated time before sample collection. (F) 3-week-old long day-grown plants were transferred to continue blue light (Blue, 35 µmol m^−2^ s^−1^) for 16 hr, and then transferred to red light (20 µmol m^−2^ s^−1^) for the indicated time.
(TIF)

Table S1 Oligonucleotide primers used in this work.
(DOCX)
