# Supplementary figures and images for: Multiple bHLH Proteins form Heterodimers to Mediate CRY2-Dependent Regulation of Flowering-Time in Arabidopsis
Source: PLoS Genet. 2013 Oct 10;9(10):e1003861. doi: 10.1371/journal.pgen.1003861 (PMC3794922; doi:10.1371/journal.pgen.1003861)

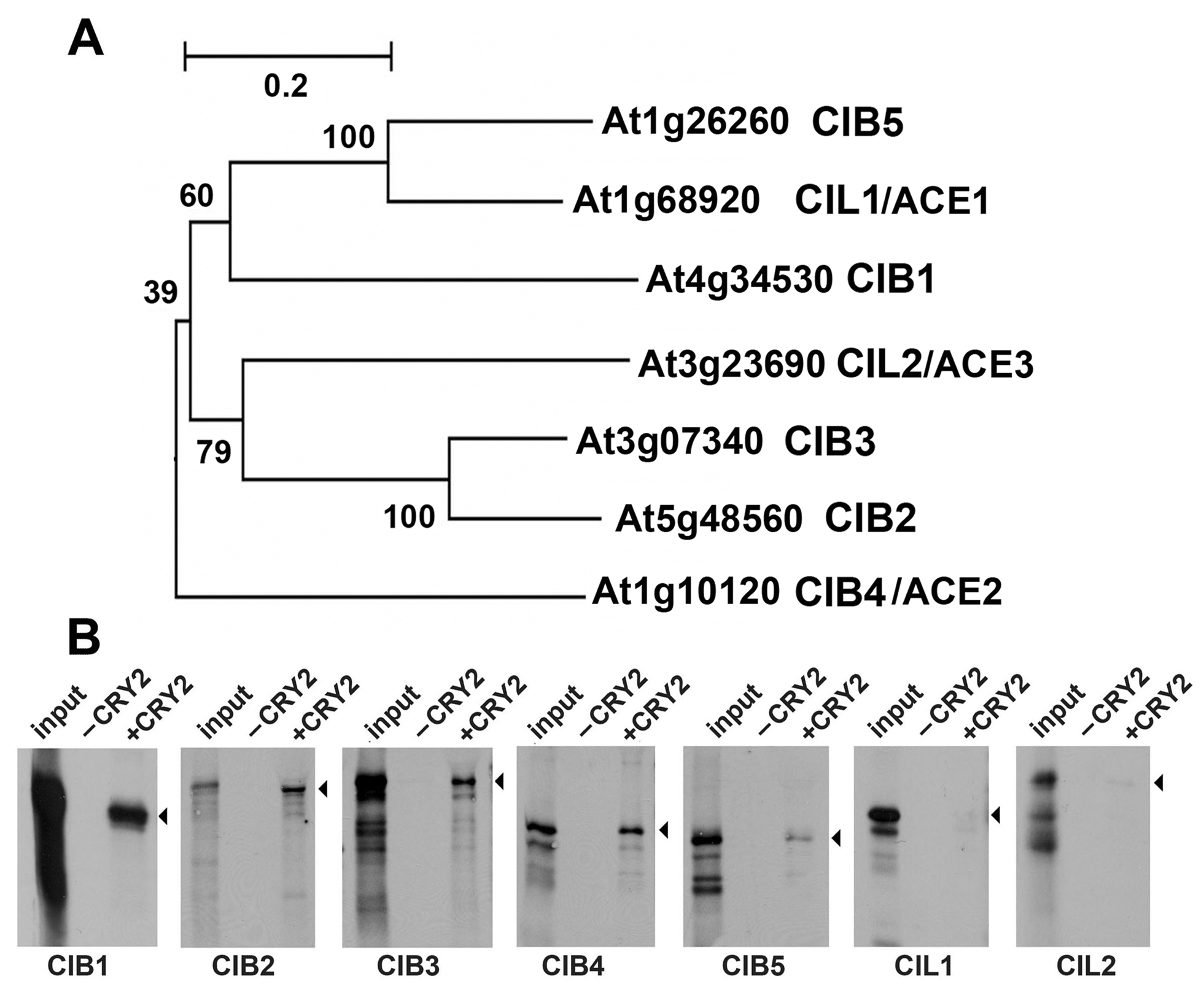

Supplement: Figure S1 — Multiple bHLH proteins interact with CRY2. (A) Neighbor-joining phylogenetic analysis (MEGA4) showing phylogenetic relationship of CIB1 and other CIB family members. The bootstrap values (1000 replicates) are indicated. The scale bar indicates substitution per site. (B) In vitro pull-down experiment showing CIB-CRY2 interactions, and the lack of CIL-CRY2 interactions. CRY2 protein expressed and purified from insect cells was incubated with the S35 labled CIB proteins prepared by the in vitro transcription/translation reactions (TnT, Promega). (TIF) [file pgen.1003861.s001.tif]

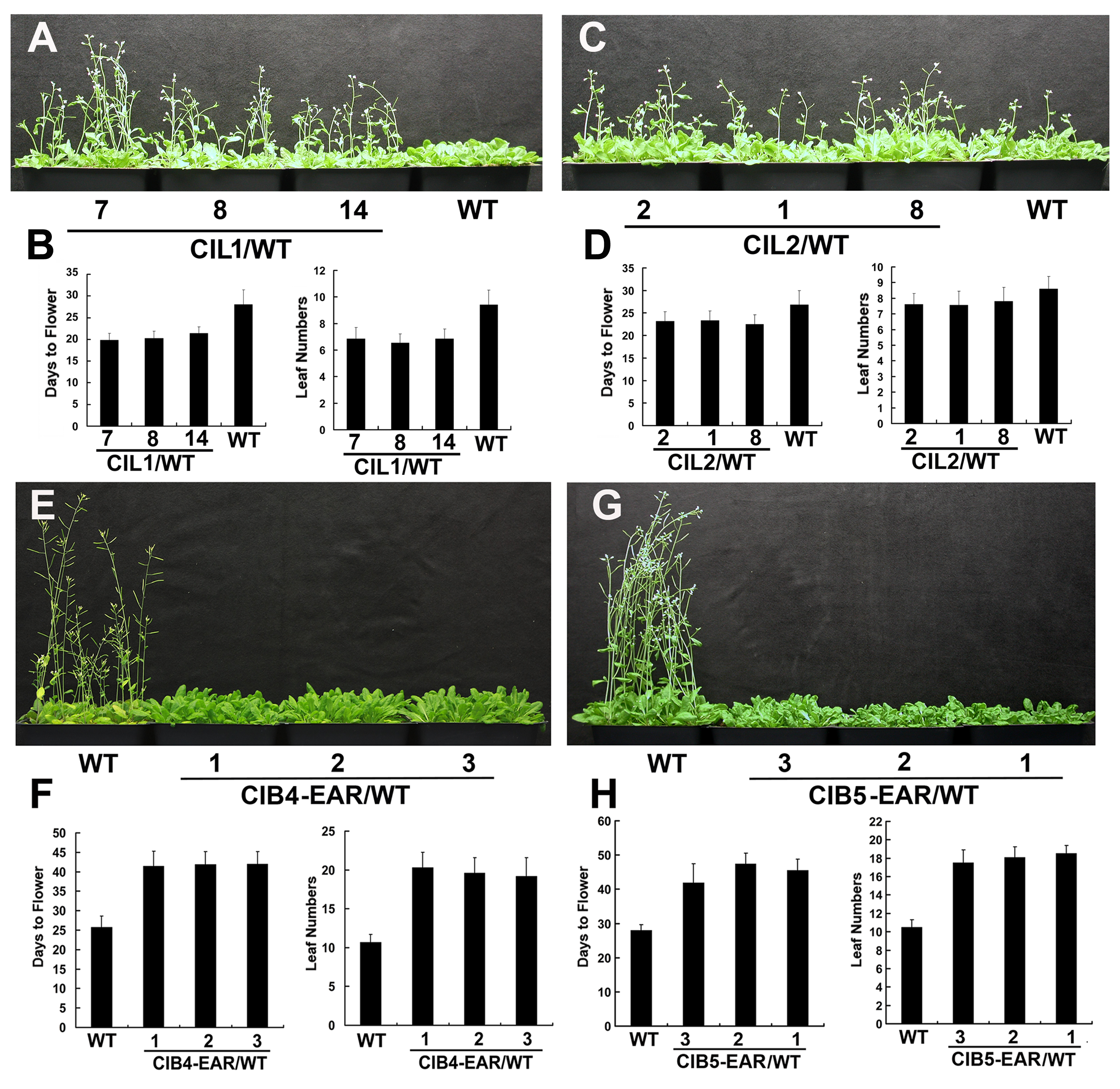

Supplement: Figure S2 — CIB4, CIB5 and CIL1 but not CIL2 promote flowering. Flowering phenotype in long day. Plants expressing the 35S::Myc-CIL1 (CIL1/WT) or 35S::Myc-CIL2 (CIL2/WT) in the wild-type background (A–D) were grown in long-day photoperiod (16 hL/8 hD) for 23 days when the pictures were taken. CIB4-EAR (E–F), CIB5-EAR (G–H) plants and the WT control were grown in long day for 33 days when the pictures were taken. The quantitative flowering times measured as days to flower and the number of rosette leaves at the day floral buds became visible, and the standard deviations (n≥20) are shown. (TIF) [file pgen.1003861.s002.tif]

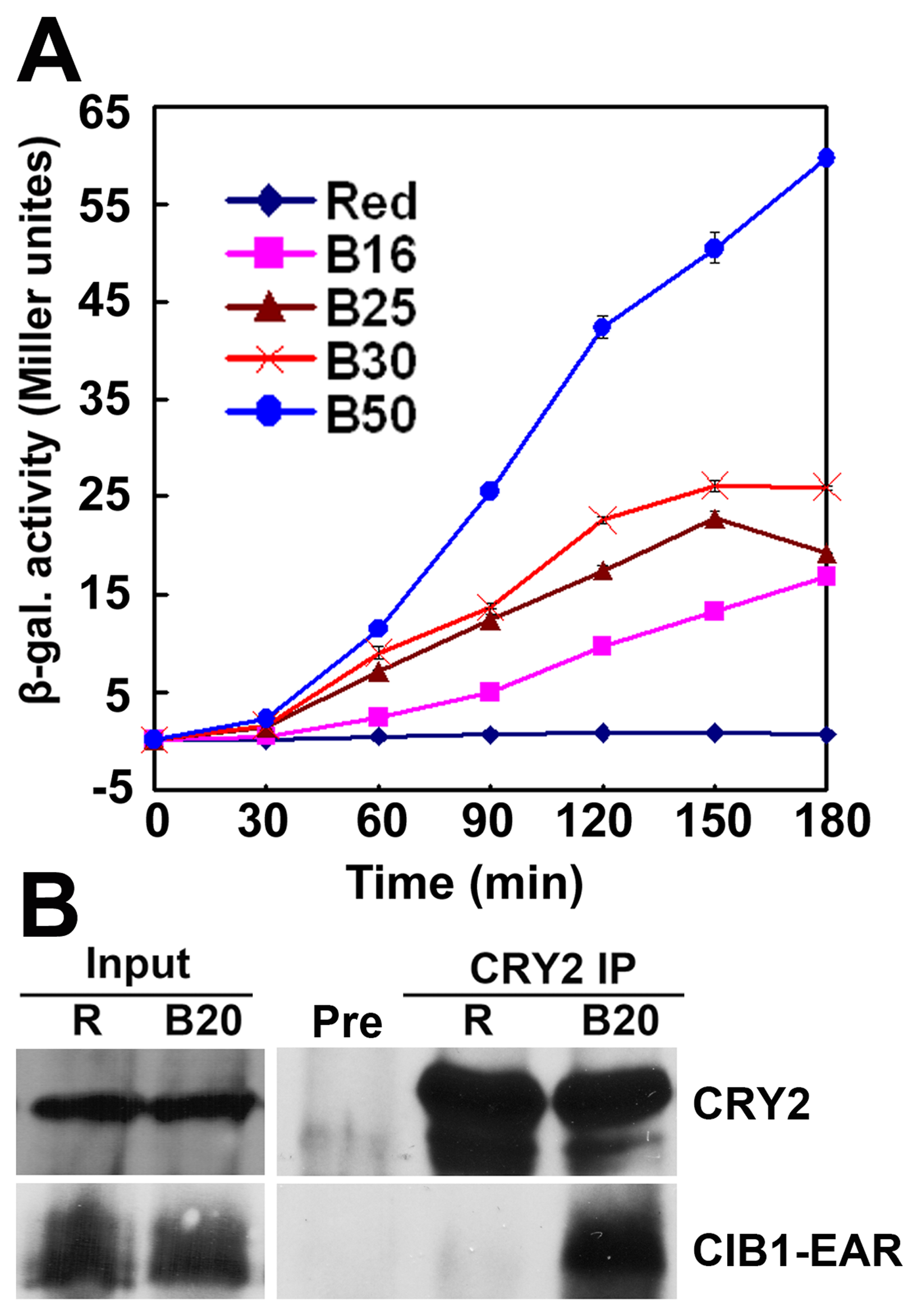

Supplement: Figure S3 — CIB1-EAR interacts with CRY2 in a blue light dependent manner. (A) β-gal assays of yeast cells expressing CIB1-EAR and CRY2 proteins irradiated with red light (R18, 18 µmol m−2 s−1) or blue light (B16 to B50, 16 to 50 µmol m−2 s−1) for the durations indicated. (B) co-IP experiment showing the blue light-dependent CRY2-CIB1EAR complex in vivo. 7-day-old seedlings expressing 35S::Myc-CIB1-EAR was grown in red light, pre-treated in MG132, and transferred to blue light (20 µmol m−2 s−1)(B20), and the IP products of the anti-CRY2 antibody were analyzed by immunoblot probed with anti-CRY2 (CRY2) or anti-Myc (CIB1) antibodies. (TIF) [file pgen.1003861.s003.tif]

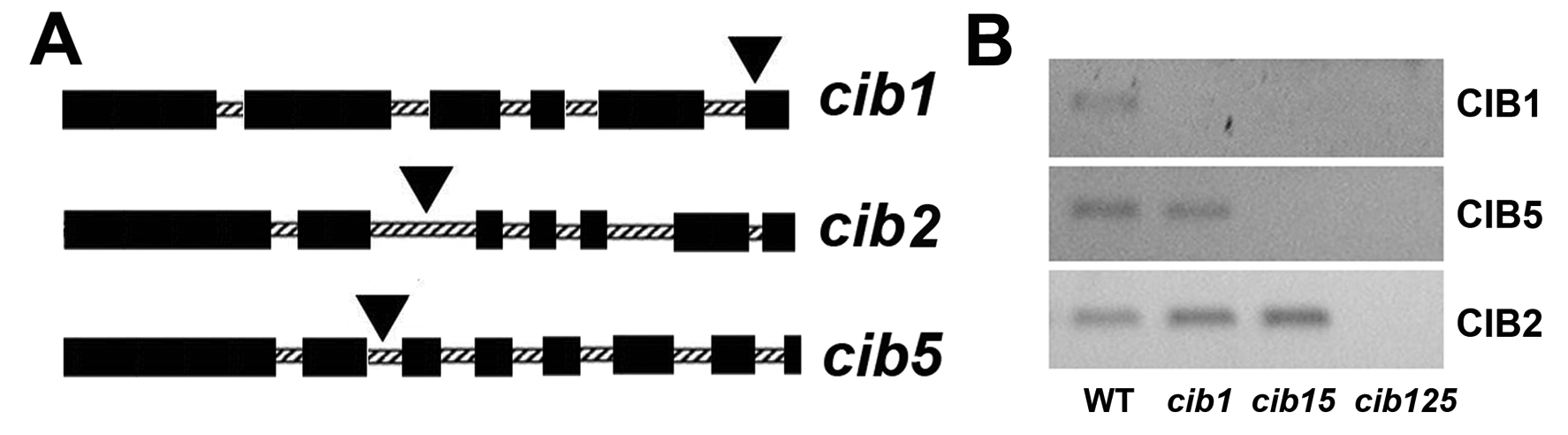

Supplement: Figure S4 — Analysis of the cib1cib2cib5 triple mutant. (A) Schematic illustrating the genomic structures of CIB1, CIB2, and CIB5 and the locations of the T-DNA insertions. Black boxes and striped boxes indicate exons and introns, respectively. T-DNA insertion sites are indicated by triangles. (B) RT-PCR analysis of CIB1, CIB2, CIB5 and Actin2 transcript abundance in wild-type (WT), cib1, cib15 and cib125 triple mutant lines. Actin2 was used as an internal control. Data shown represent one of three independent assays that gave the same results. (TIF) [file pgen.1003861.s004.tif]

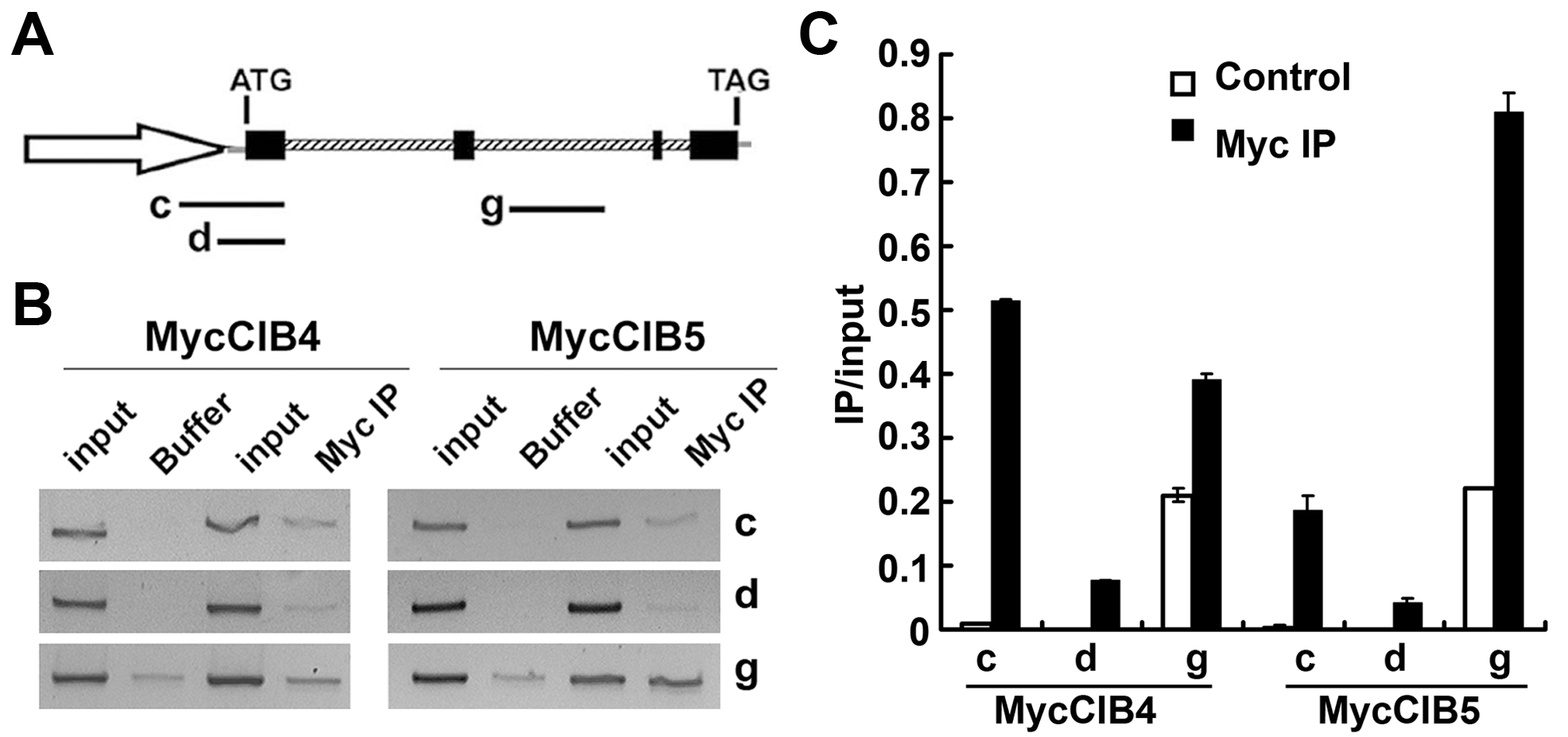

Supplement: Figure S5 — ChIP-PCR showing interaction of CIB4 and CIB5 with chromatin regions of the FT gene. (A) A diagram depicting the putative promoter (arrow), 5′ UTR (grey line), exons (black boxes), introns (dashed boxes), 3′ UTR (grey line) of the FT gene. Black solid lines depict the DNA regions that were amplified by ChIP-PCR using the indicated primer sets. (B) Representative results of the ChIP-PCR assays. Chromatin fragments (∼500 bp) were prepared from 7-day-old transgenic seedlings expressing 35S::Myc-CIB4 or 35S::Myc-CIB5, immunoprecipitated by the anti-Myc antibody, and the precipitated DNA PCR-amplified using the primer pairs indicated. Input: PCR reactions using the samples before immunoprecipitation. (C) ChIP-PCR results for the primer pairs that were repeated at least three times were quantified by normalization of the Myc-IP signal with the corresponding input signal (IP/input). The standard deviations (n≥3) are shown. (TIF) [file pgen.1003861.s005.tif]

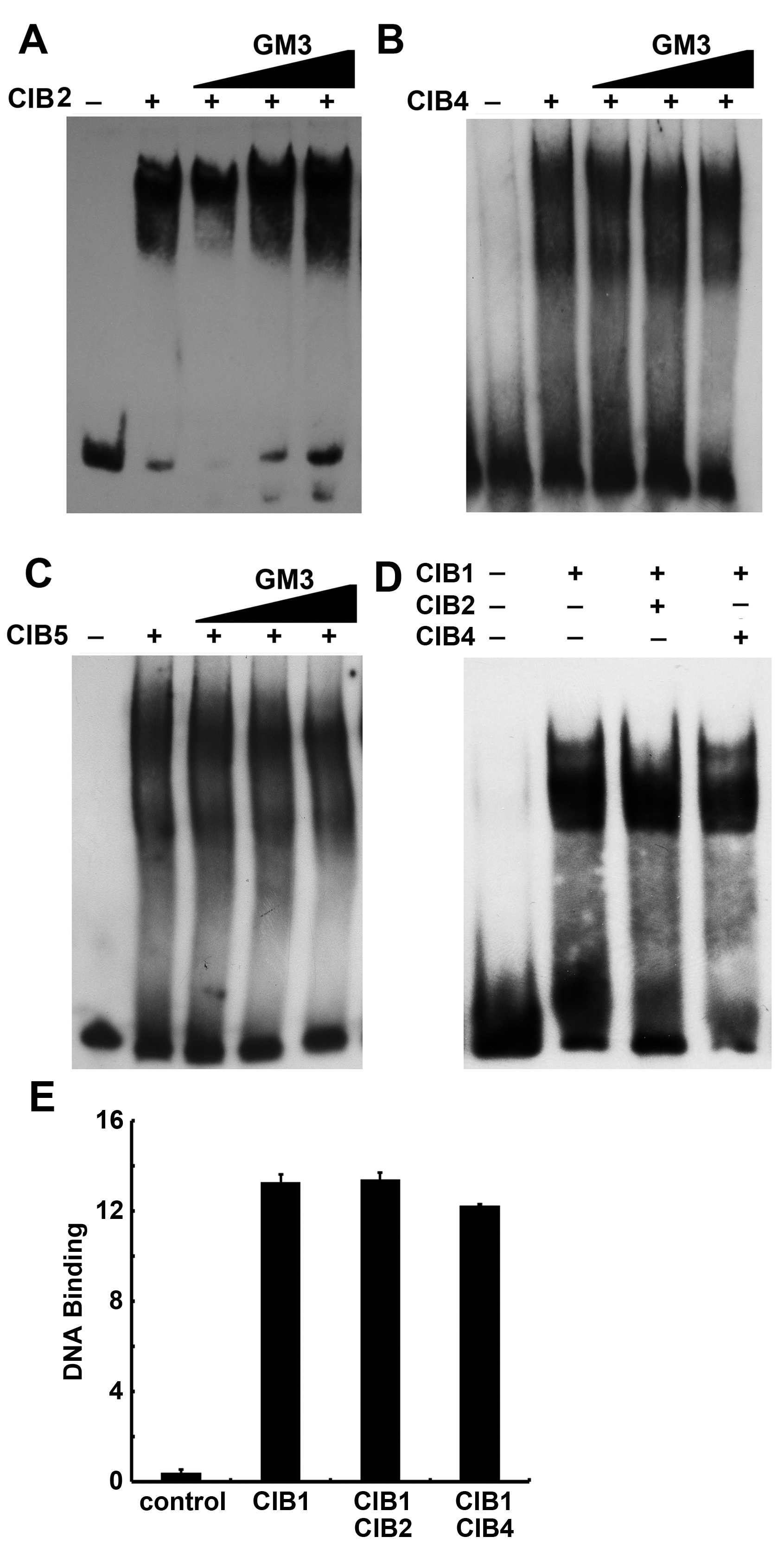

Supplement: Figure S6 — CIB2, CIB4, CIB5 bind to G-box specificly in vitro. (A–C) A competitive EMSA showing interaction of CIB2, CIB4, CIB5 with the DIG-labeled G-box (canonical E-box), and lack of a strong competition by the mutant G-box (Gm3: CAAGTG). Black wedges represent increasing amount of competitors (12.5×, 25×, 50× in molar excess). (D) An EMSA experiment showing association of the CIB1CIB2 or CIB1CIB4 heterodimers, and also CIB1 monomer, with the G-box DNA (CACGTG, canonical E-box). The indicated CIB proteins were expressed and purified from E. coli, and incubated with the labeled oligonucleotide containing the G-box (canonical E-box). (E) A semi-quantitative analysis of DNA binding of the EMSA shown in (D). The film shown in (D) were scanned, and analyzed by Image J software. (TIF) [file pgen.1003861.s006.tif]

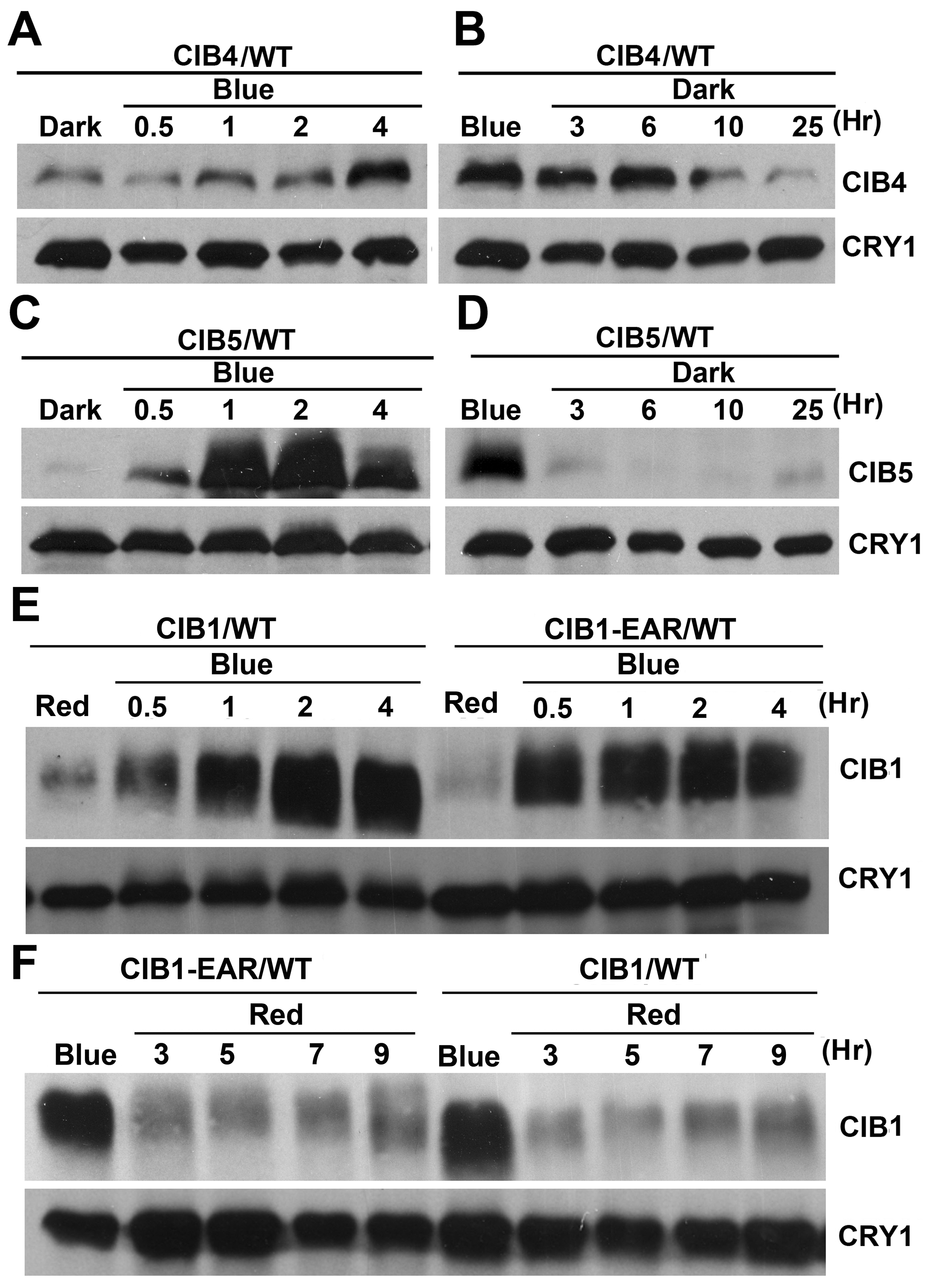

Supplement: Figure S7 — CIB4, CIB5 and CIB1-EAR are degraded in the absence of blue light. (A–D) Immunoblot experiments showing the light regulation of CIB4, CIB5 protein expression in transgenic plants expressing the 35S::Myc-CIB4 or 35S::Myc-CIB5 transgene. Samples were fractionated by 10% SDS-PAGE, blotted, and probed by the anti-Myc antibody, stripped and re-probed with the anti-CRY1 antibody to indicate relative loading of the samples. In the first experiment (A, C), 3-week-old long day-grown (16 hL/8 hD) plants were transferred to dark for 16 hr, and then transferred to blue light (35 µmol m−2 s−1) for the indicated time before sample collection. In the second experiment (B, D), 3-week-old long day-grown plants were transferred to continuous blue light (Blue, 35 µmol m−2 s−1) for 16 hr, and then transferred to dark for the indicated time. (E–F) Immunoblot experiments showing the light regulation of not only CIB1 but also CIB1-EAR protein expression in transgenic plants expressing the 35S::Myc-CIB1 or 35S::Myc-CIB1-EAR transgene. (E) 3-week-old long day-grown (16 hL/8 hD) plants were transferred to red light (20 µmol m−2 s−1) for 16 hr, and then transferred to blue light (35 µmol m−2 s−1) for the indicated time before sample collection. (F) 3-week-old long day-grown plants were transferred to continue blue light (Blue, 35 µmol m−2 s−1) for 16 hr, and then transferred to red light (20 µmol m−2 s−1) for the indicated time. (TIF) [file pgen.1003861.s007.tif]
