# Supplementary material for: Multiple bHLH Proteins form Heterodimers to Mediate CRY2-Dependent Regulation of Flowering-Time in Arabidopsis
Source: PLoS Genet. 2013 Oct 10;9(10):e1003861. doi: 10.1371/journal.pgen.1003861 (PMC3794922; doi:10.1371/journal.pgen.1003861)
Supplement: Table S1 — Oligonucleotide primers used in this work. (DOCX) [file pgen.1003861.s008.docx]

Table S1. Oligonucleotide primers used in this work

| Assays | Destination products | Templet | Primer name | Primer sequence |
| --- | --- | --- | --- | --- |
| Transformation | pEGAD-Myc-VP16-CIB1  pEGAD-Myc-CIB1-EAR  pEGAD-Myc-CIB2  pEGAD-Myc  CIB3  pEGAD-Myc  CIB4  pEGAD-Myc  CIB5  pEGAD-Myc  CIL1  pEGAD-Myc  CIL2  pEGAD-Myc-CIB4-EAR  pEGAD-Myc-CIB5-EAR  pEGAD-LUC | VP16  CIB1  CIB2  CIB3  CIB4  CIB5  CIL1  CIL2  CIB4  CIB5  LUC | VP16F  VP16R  CIB1F  CIB1EAR  48560F  48560R  07340F  07340R  10120F  10120R  26260F  26260R  68920F  68920R  23690F  23690R  10120F  CIB4EAR  26260F  CIB5EAR  LUC-F  LUC-R | 5’-GAATTCACCGCCCCCATTACCGACG-3’  5’-GAATTCCCCCCCAAAGTCGTCAATG-3’  5’-GAATTCATGAATGGAGCTATAGGAG-3’  5’-CTCGAGTTAAGCGAAACCCAAACGGAGTTCTAGATCCAGATCGAGAAGCTTAACTCCTAAATTGCCATAG-3’  5’-CCCGGGATGGACAACGAGCTG-3’  5’-CCGCTCGAGTCAAAGCTCAATTTTC-3’  5’-CTCGAGATGGAGAACGAGCTGTTTATG-3’  5’-CCGCTCGAGTCAAAGTTCAGCTTTCATG-3’  5’-GGAATTCATGGGTGGTGGAGTAATG-3’  5’-CCGCTCGAGCTAGAGCTCAGGTTTC-3’  5’-GGAATTC ATGAGTGACA AAGACG-3’  5’-CCGCTCGAGCTACGGCTCCACCTTC-3’  5’-CCCGGGATGGATTTAAGTGCGAAAG-3’ 5’-CCGCTCGAGTCATGGCTCAACCTTC-3’  5’-CCCGGGATGAACATGGACAAGG-3’  5’-CCGCTCGAGCTATGGTTCAAGCTTC-3’  5’-GGAATTCATGGGTGGTGGAGTAATG-3’  5’-CCGCTCGAGTTAAGCGAAACCCAAACGGAGTTCTAGATCCAGATCGAGCCCGGG GAGCTCAGGTTTCAATCGAC-3’  5’-GGAATTC ATGAGTGACA AAGACG-3’  5’-CCGCTCGAGTTAAGCGAAACCCAAACGGAGTTCTAGATCCAGATCGAGCCCGGG CGGCTCCACCTTCATGTC-3’  5’-ACCGGTATGGAAGACGCCAAAAAC-3’  5’-G GAATTC CACGGCGATCTTTCCG-3’ |
| Transfromation | pCAMBIA1300 | DNA | CIB2PF  CIB2PR  CIB4PF  CIB4PR  CIB5PF  CIB5PR | 5’-CTGCAGGAGCTCTTACAAACCAGAATATTTACAAACAGTG-3’  5’-GGATCCACCGGTTTTCTCTCTAATCTTTGAATAAGAAGAAG-3’  5’-CTGCAGGAGCTCTTTGTTTAACCTTGCTTTTGTAAGAAG-3’  5’-TCTAGAACCGGTTCCTCTGCTTCAACAACAACCA-3’  5’-CTGCAGGAGCTCTATTTTTTAGACGATCGGGTAAATA-3’  5’-TCTAGAACCGGTTCTCTTAAAAGTAGTAAAGCCCCAA-3’ |
| Co-localization | PC131-CRY2-mCherry  PC131-CIB1-mCherry  PC131-CIB2-YFP  PC131-CIB4-YFP  PC131-CIB5-YFP | CRY2  CIB1  CIB2  CIB4  CIB5 | CRY2F  CRY2R  CIB1F  CIB1R  CIB2F  CIB2R  CIB4F  CIB4R  CIB5F  CIB5R | 5’-ACGCGTCGAC  ATGAAGATGGACAAAAG-3’  5’-GGACTAGT  TTTGCAACCATTTT-3’  5’-ATGAATGGAGCTATAGGA-3’  5’-AACTCCTAAATTGCC-3’  5’-ACGCGTCGAC ATGGACAACGAGCTG-3’  5’-GGACTAGT  AAGCTCAATTTTCATGT-3’  5’-ACGCGTCGAC ATGGGTGGTGGAGTAATG-3’  5’-GGACTAGT  GAGCTCAGGTTTCAAT-3’  5’-ACGCGTCGAC  ATGAGTGACA AAGACG-3’  5’-GGACTAGT  CGGCTCCACCTTCATGT-3’ |
| BiFC | pCCFP-CRY2  pCCFP-CIB1  pNYFP-CIB2  pNYFP-CIB4  pNYFP-CIB5 | CRY2  CIB1  CIB2  CIB4  CIB5 | CRY2F  CRY2R  CIB1F  CIB1R  CIB2F  CIB2R  CIB4F  CIB4R  CIB5F  CIB5R | 5’GGGGACAAGTTTGTACAAAAAA  CAGGCTTCATGAAGATGGACAAAA  AGAC-3’  5’GGGGACCACTTTGTACAAGAAA  CTGGGTCCTATCATTTGCAACCATT  TTTTCC-3’  5’GGGGACAAGTTTGTACAAAAAA  CAGGCTTCATGAATGGAGCTATAG  GAG-3’  5’GGGGACCACTTTGTACAAGAAA  CTGGGTCTCAAACTCCTAAATTGCC  ATAG-3’  5’-CACC ATGGACAACGAGCTG-3’  5’- TCAAAGCTCAATTTTC-3’  5’-CACC ATGGGTGGTGGAGTAATG-3’  5’-CTAGAGCTCAGGTTTC-3’  5’-CACC ATGAGTGACA AAGACG-3’  5’-CTACGGCTCCACCTTC-3’ |
| Dual-LUC | pGreen-0800-FTp | FT promoter | FTpF_2K_  FTpR_UTR_ | TGTCAATGCTTACTATATCATCTTA  TGTTTTACTTGTTTTTGTTTCTGCT |
| EMSA |  |  | GboxF,  GboxR  MG3F  MG3R  FT-EboxF  FT-EboxR  FTE-MF  FTE-MR | 5’AGGAGAGTGGGCCACGTGCGCTCTTTTGCATTC-3’  5’GAAGAATGCAAAAGAGCGCACGG GCCCACTCT-3’  5’AGGAGAGTGGGCCAAGTGCGCTCTTTTGCATTC-3’  5’GAAGAATGCAAAAGAGCGCACTT  GGCCCACTCT-3’  5’-AGTGGCTACCAAGTGGGAGATATA-3  5’- TATATCTCCCACTTGGTAGCCACT-3  5’-AGTGGCTACAAAAAAGGAGATATA-  5’-TATATCTCCTTTTTTGTAGCCACT-3’ |
| Protein  expression | pCold-TF-  CIB1  pCold-TF-  CIB2  pCold-TF-  CIB4  pCold-TF-  CIB5 | CIB1  CIB2 | cibPF,  cibGEXR  CIB2F1  48560R  10120F  10120R  26260F  26260R | 5’-GAATTCATGAATGGAGCTATAGGAG-  5’-CTCGAGTCAAACTCCTAAATTGCC-3  5’-GGAATTCCATATGATGGACAACGAGCTG-3’  5’-CCGCTCGAGTCAAAGCTCAATTTTC-3’  5’-GGAATTCATGGGTGGTGGAGTAATG-3’  5’-CCGCTCGAGCTAGAGCTCAGGTTTC-3’  5’-GGAATTC ATGAGTGACA AAGACG-3’  5’-CCGCTCGAGCTACGGCTCCACCTTC-3’ |
| RT-PCR |  | CIB1  CIB2  CIB4  CIB5 | CIB1F  CIB1R  CIB2F  CIB2R  CIB4F  CIB4R  CIB5F  CIB5R | 5’-GAATTCATGAATGGAGCTATAGGAG-  5’-AAGCTTTCAAACTCCTAAATTGCC-3’  5’-GATTTTCATCTTCCGTAGTCC-3’  5’-GTTATGTACACCCATCAATCC-3’  5’-GGAATTCATGGGTGGTGAGAGTAATG-3’  5’-CCGCTCGAGCTAGAGCTCAGGTTTC  5’-TGAGTGACAAAGACGAGTTTGC-3’  5’-AGAAACATCTACGGCTCCACC-3’ |
| Chip |  | c  d  g | FTcF  FTcR  FTdF  FTdR  FTgF  FTgR | 5’-TTATGATTTCACCGACCC-3’  5’-CAAGCCATTAGTCACCTCTC-3’  5’-CAATCAACACAGAGAAACCAC-3’  5’-AGGTCTTCTCCACCAATCTC-3’  5’-AAAAAGCCCACACCCAAG-3’  5’-AAACCTAGTCCTGCTCACTTC-3’ |
| Q-PCR  Q-PCR for Chip |  | a  c  g  NC | QFTF  QFTR  QACT2F  QACT2R  QFTaF  QFTaR  QFTcF  QFTcR  QFTgF  QFTgR  QCOF,  QCOR, | 5’-CAACCCTCACCTCCGAGAATAT3’  5’-TTGCCAAAGGTTGTTCCAGTT-3’  5’-GTGGATTCCAGCAGCTTCCAT-3’  5’-GCTGAGAGATTCAGATGCCCA-3’  5’- ATATATCGGATTAAATCAAAAAACA-  5’-ATGTATGCATTTTTAAATATTGGAC-  5’- GACGACAATGTGTGATGTACG-3’  5’- GTATCATAGGCATGAACCCTCT-3’  5’-CAACCCTCACCTCCGAGAATAT-3’  5’-TTGCCAAAGGTTGTTCCAGTT-3’  5’-CCGGGTCTGCGAGTCATG-3’  5’-GGCATCATCTGCCTCACACA-3’ |
